# Visible Light‐Driven Heterojunction Array Based on Type‐I In_2_S_3_/In_2_O_3_ for Selective Multi‐Gas Discrimination

**DOI:** 10.1002/smll.202506056

**Published:** 2025-11-18

**Authors:** Gi Baek Nam, Jaekwon Ko, Seungwook Choi, Sungkyun Choi, Jin Wook Yang, Hee Ryeong Kwon, Yeong Jae Kim, Jihwan Kwon, Jongchul Jeon, Ansoon Kim, Young‐Seok Shim, Seung‐Wook Baek, In‐Hyeok Park, Ho Won Jang, Ki Chang Kwon

**Affiliations:** ^1^ Department of Materials Science and Engineering Research Institute of Advanced Materials Seoul National University Seoul 08826 Republic of Korea; ^2^ Division of Chemical and Material Metrology Korea Research Institute of Standards and Science Daejeon 34133 Republic of Korea; ^3^ Graduate School of Analytical Science and Technology Chungnam National University Daejeon 34134 Republic of Korea; ^4^ Strategic Technology Research Institute Korea Research Institute of Standards and Science Daejeon 34133 Republic of Korea; ^5^ Department of Applied Measurement Science University of Science and Technology Daejeon 34113 Republic of Korea; ^6^ School of Energy Materials & Chemical Engineering Korea University of Technology and Education Cheonan 31253 Republic of Korea; ^7^ Advanced Institute of Convergence Technology Seoul National University Suwon 16229 Republic of Korea; ^8^ Department of Chemical Engineering Chung‐Ang University Seoul 06974 Republic of Korea

**Keywords:** array, chemoresistive gas sensor, heterojunction, indium sulfide, light activation

## Abstract

Visible light‐activated chemoresistive gas sensors offer a promising solution for minimizing power consumption, preventing material degradation, and enabling room temperature operation. However, their lower photon energy compared to UV light results in slower recovery and reduced sensitivity to NO_2_, while detecting gases like volatile organic compounds and amines remains challenging. This work presents a visible light‐driven gas sensor array based on type‐I In_2_S_3_/In_2_O_3_ heterostructure. The In_2_S_3_ layer is uniformly deposited on In_2_O_3_ nanorods, forming type‐I band alignment. Under blue light illumination, photoexcited electron‐hole pairs migrate to the In_2_S_3_ surface, enhancing surface reactivity and enabling 56 times higher NO_2_ response than pristine In_2_O_3_, with excellent selectivity, reliability, and humidity stability. Noble metal (Pd, Pt, and Au) decoration on the In_2_S_3_‐In_2_O_3_ array also allows truly selective detection of NO_2_, NH_3_, C_2_H_5_OH, and H_2_, which has not been reported previously for light‐activated gas sensors. This work introduces a new strategy to optimize visible light‐driven gas detection, advancing electronic nose technologies.

## Graphical abstract

Visible light activation has been explored for low‐temperature operation, prevention of material degradation, and safe gas sensing. A finely tuned type‐I band junction within the In_2_S_3_‐In_2_O_3_ heterostructure promotes efficient transfer of photogenerated excitons to the In_2_S_3_ surface, thereby maximizing surface reactivity under blue light illumination. Noble metal‐decorated In_2_S_3_‐In_2_O_3_ heterostructure demonstrated selective detection of NO_2_, NH_3_, H_2_, and C_2_H_5_OH.

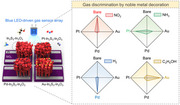

## 1 Introduction

Chemical sensors have gained significant attention for diverse applications in medical diagnostics, food quality monitoring, smart agriculture, and detecting harmful chemicals.^[^ ^1^, ^2^, ^3^ ^]^ The integration of sensor systems with Internet of Things technologies and rapid advancements in artificial intelligence (AI) enables real‐time environmental monitoring and facilitates smart home ecosystems.^[^ ^4^ ^]^ This combination promises to enrich our daily lives by providing more intelligent, responsive environments. Specifically, chemoresistive gas sensors are particularly promising for chemical sensor platforms due to their simple device architecture, rapid response times, and resistance‐based signal readouts, which enable easy electronic integration.^[^ ^5^, ^6^ ^]^ However, commercially available metal oxide semiconductor‐based chemoresistive gas sensors require high operating temperatures (200–400 °C), leading to performance degradation from heating, high power consumption, and limiting their use in wearable or flexible devices.

To overcome these limitations of conventional gas sensors, low‐temperature operable light‐activated chemoresistive gas sensors have emerged as a promising alternative.^[^ ^7^, ^8^, ^9^, ^10^ ^]^ Upon light illumination, exciton pairs are generated, and adsorbed oxygen at the surface is activated, thereby lowering the activation energy required for gas reactions.^[^ ^11^ ^]^ While ultraviolet (UV) illumination is adequate for activation, its high photon energy poses health risks, including skin cancers,^[^ ^12^ ^]^ burns,^[^ ^13^ ^]^ and aging.^[^ ^14^ ^]^ In contrast, visible‐light‐driven gas sensors have attracted considerable attention due to their inherent safety and biocompatibility. Nonetheless, owing to the weak intensity of visible light, activating metal oxide semiconductors with large band gaps remains a significant challenge. Many studies have reported limited success, including low NO_2_ responses, slow recovery, and an inability to detect other gases like amines, volatile organic compounds (VOCs), and hydrogen (H_2_), which are relevant to toxicity, industrial environments, and flammability. For example, Eom et al. reported blue‐light‐activated SnS_2_ nanoflowers with weak NO_2_ response and detection limited to NO_2_.^[^ ^15^ ^]^ Additional strategies, including plasmonic resonance via Au nanoparticle decoration,^[^ ^11^, ^16^, ^17^, ^18^, ^19^ ^]^ and band gap tuning,^[^ ^20^, ^21^ ^]^ have been explored, yet multi‐gas detection under visible‐light operation has not been demonstrated.

Meanwhile, band alignment strategies have proven successful in enhancing photo‐efficiency in various optoelectronic devices, including light‐emitting diodes (LED),^[^ ^22^ ^]^ photoelectrochemical (PEC) water splitting,^[^ ^23^ ^]^ and photodetectors.^[^ ^24^ ^]^ For instance, type‐I quantum‐well heterojunctions in AlGaAs/GaAs/AlGaAs structures for charge localization to maximize luminance efficiency,^[^ ^25^ ^]^ while type‐II heterostructures like In_2_O_3_/BiVO_4_,^[^ ^26^ ^]^ TiO_2_/BiVO_4_,^[^ ^27^ ^]^ and Co_3_O_4_/TiO_2_,^[^ ^28^ ^]^ are widely used in PEC water splitting for effective charge separation. Despite the critical role of photoexcited charge carriers in light‐driven gas sensors, the systematic application of band alignment in heterojunctions to enhance reactivity in this field remains largely underexplored.

**Scheme 1:**
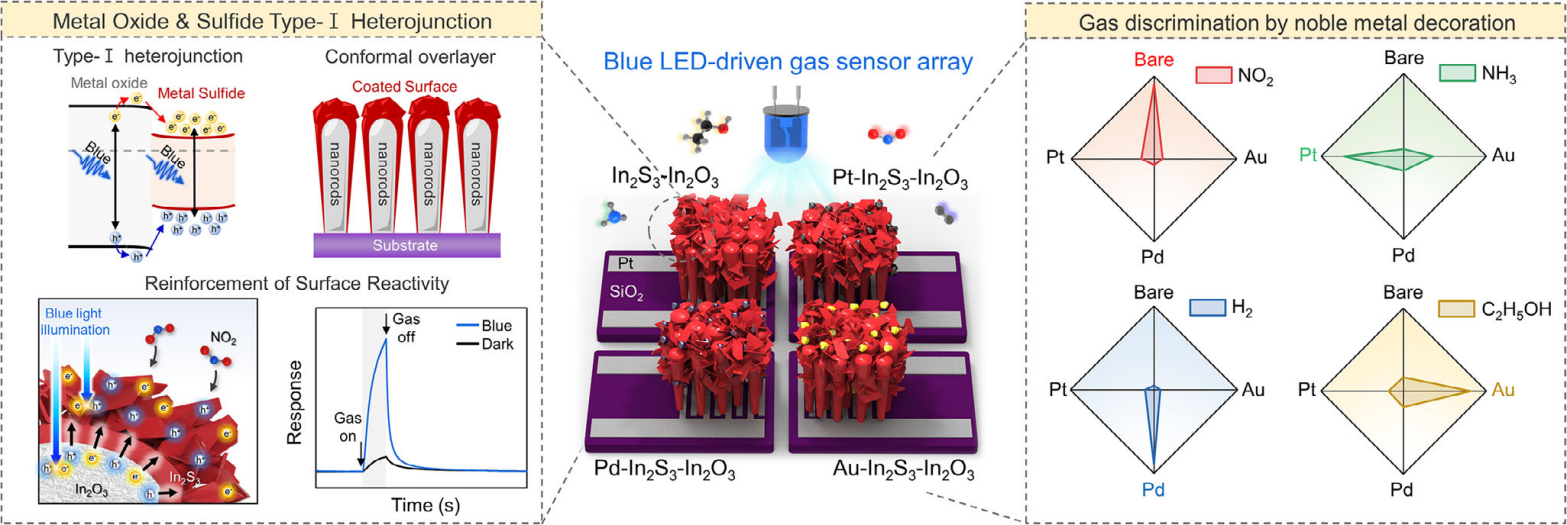
Schematic illustration of the visible‐light activation strategy by type‐I heterojunction and gas discrimination using noble‐metal decorated ISO gas sensor array.

Herein, we present a visible light‐activated gas sensor array based on In_2_S_3_‐In_2_O_3_ (ISO) heterostructures engineered to form type‐I band alignment (**Scheme** **1**). Although In_2_O_3_ has a wide bandgap, it readily forms oxygen vacancies that generate sub‐band‐gap states, enabling visible‐light absorption.^[^ ^29^, ^30^ ^]^ In addition, the high electron mobility of In_2_O_3_ exhibits low baseline resistance for room‐temperature operation.^[^ ^31^, ^32^ ^]^ In_2_S_3_ has a smaller bandgap in the green light spectral range, a high absorption coefficient, and excellent photoresponse.^[^ ^33^ ^]^ Structural defect sites in In_2_S_3_ also provide abundant active sites for gas interaction.^[^ ^34^ ^]^ To exploit these complementary properties and to maximize junction density, the ISO heterostructures are fabricated by a two‐step process. In_2_O_3_ nanorods (NRs) were fabricated via an electron‐beam (e‐beam) evaporator using glancing angle deposition (GLAD), followed by the uniform growth of In_2_S_3_ nanocrystals through chemical vapor deposition (CVD) methods. Under blue‐light illumination, which lies within the band gap between In_2_O_3_ and In_2_S_3_, photogenerated exciton pairs are produced in both materials. Due to the type‐I junction, photoexcited carriers from the In_2_O_3_ transfer to the In_2_S_3_ surface, significantly enhancing surface reactivity for gas interactions.^[^ ^34^, ^35^ ^]^

Gas sensing properties were evaluated by ISO heterostructures toward NO_2_ under red, green, blue, and UV illumination, with NO_2_ selected as a representative hazardous industrial emission. The ISO sensors exhibit a remarkable 56 times higher gas response to NO_2_ under blue light illumination than bare In_2_O_3_ NRs, and the highest response compared to the ISO sensors under red, green, and UV light illumination, coupled with fast recovery, high selectivity, and excellent humidity stability. Furthermore, we demonstrate a 2 × 2 sensor array comprising four gas sensors, driven by blue‐LED illumination that addresses the limitation of single sensors in detecting diverse analytes and discriminating gas mixtures. The noble metal decoration is a well‐established strategy to enhance selectivity and improve sensor performance, but most noble metal decorated gas sensor arrays require high operating temperatures to activate the noble metal effect effectively.^[^ ^36^, ^37^, ^38^ ^]^ Notably, the ISO type‐I junction facilitates the interaction of photogenerated exciton pairs with the noble metal deposited on the In_2_S_3_ surface, thereby lowering the activation energy for the detection of other analytes, including NH_3_, C_2_H_5_OH, and H_2_ at room temperature. Noble metal decorated ISO (Pd, Pt, and Au) exhibited selective detection of NH_3_, H_2_, and C_2_H_5_OH, respectively. The ISO‐based sensor array successfully discriminates gas mixtures, indicating strong potential for multi‐gas detection in hydrogen production facilities and vehicle exhaust atmospheres, where distinguishing interfering gases is essential. These findings break through the limitation of visible‐light activation and contribute to developing next‐generation, low‐power, human‐compatible sensor platforms.

## 2 Results and Discussion

### 2.1 Characterization of In_2_S_3_‐In_2_O_3_

**Figure 1:**
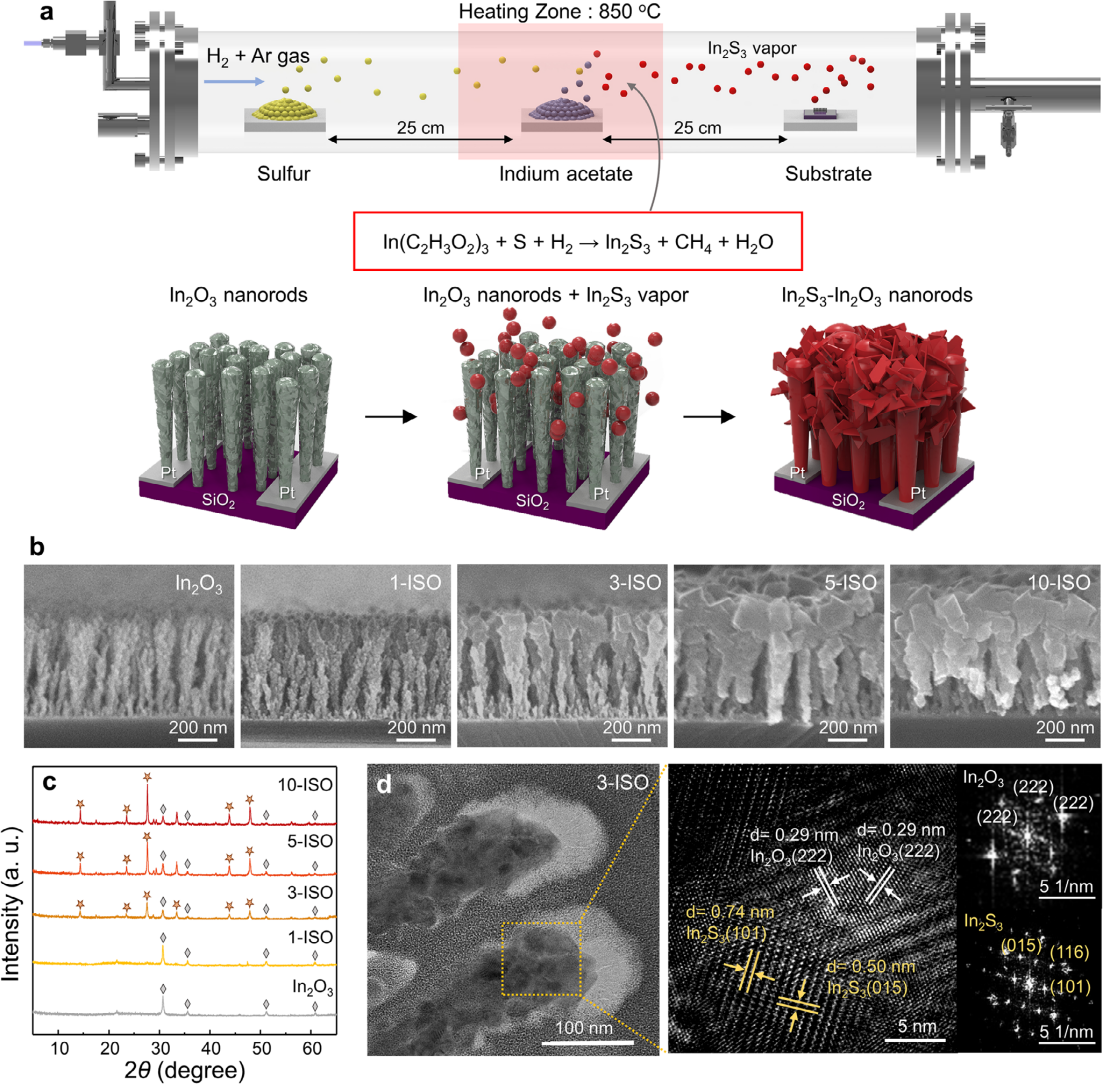
a) Schematic illustration of the direct growth of In_2_S_3_ on In_2_O_3_ nanorods via the CVD process. b) Cross‐sectional SEM images of 3‐ISO nanorods with different In_2_S_3_ evaporation times on In_2_O_3_ nanorods. c) XRD spectrum of ISO with different In_2_S_3_ evaporation times on In_2_O_3_ nanorods. d) TEM image and HRTEM image with FFT patterns of 3‐ISO.

The ISO was synthesized through a two‐step process involving e‐beam deposition of In_2_O_3_ NRs followed by CVD growth of In_2_S_3_. Initially, In_2_O_3_ NRs were deposited onto Pt‐interdigitated electrodes (Pt‐IDEs) using the GLAD method for the self‐shadowing effect that promoted the growth of vertically aligned In_2_O_3_ NRs (Figure S1, Supporting Information).^[^ ^39^ ^]^ These NRs exhibited a porous structure with a thickness of ≈500 nm. Subsequently, In_2_S_3_ nanocrystals were deposited on the surface of In_2_O_3_ NRs via the CVD process using the sulfur and indium acetate [In(CH_3_COO)_3_] powders (**Figure** **1**a). The lower boiling point of indium acetate, compared to In_2_O_3_ powder, facilitated the formation of uniform In_2_S_3_ thin films. The sulfur and indium acetate vapors formed In_2_S_3_, as described in Equation (1).

(1) $$2\;\mathrm{In}{\left(C_{2}H_{3}O_{2}\right)}_{3}+3\;S+27\;H_{2}\to In_{2}S_{3}+12\;CH_{4}+12\;H_{2}O$$

The resultant In_2_S_3_ vapor was deposited onto the surface of the In_2_O_3_ nanorods, forming the ISO heterostructures. Cross‐sectional scanning electron microscope (SEM) images illustrate the progression of In_2_S_3_ deposition as a function of CVD growth time (Figure 1b). Samples were designated as 1‐ISO, 3‐ISO, 5‐ISO, and 10‐ISO, corresponding to growth times of 1, 3, 5, and 10 min, respectively. The bare In_2_O_3_ NRs exhibited a 1D structure with an average length of 500 nm. With increasing In_2_S_3_ growth time, minimal morphological changes were observed in 1‐ISO samples, while surface coverage of In_2_S_3_ on In_2_O_3_ NRs became evident in the 3‐ISO samples. For the 10‐ISO sample, the In_2_S_3_ layer was fully deposited, forming a continuous film over the In_2_O_3_ NRs. This was consistent with the observed decrease in porosity as a function of the In_2_S_3_ deposition time, as shown in top‐view SEM images and void fraction area (Figure S2, Supporting information).

X‐ray diffraction (XRD) analysis was performed to determine the phase composition and crystallinity of bare In_2_O_3_ NRs and ISO heterostructures as a function of the In_2_S_3_ layer growth time (Figure 1c). Before the deposition of the In_2_S_3_, the diffraction pattern was dominated by peaks corresponding to highly crystalline In_2_O_3_ NRs, consistent with the In_2_O_3_ cubic structure. As the In_2_S_3_ deposition time increased, peaks associated with In_2_S_3_ became progressively intense. From the 3‐ISO sample, In_2_S_3_ diffraction peaks dominated, while the intensity of the In_2_O_3_ peaks decreased, indicating that In_2_S_3_ nearly fully covered the In_2_O_3_ surface. The In_2_S_3_ deposited directly onto a SiO_2_ wafer exhibited distinct diffraction peaks corresponding to specific crystalline facets of In_2_S_3_ (Figure S3, Supporting information). In contrast, In_2_S_3_ deposited on In_2_O_3_ NRs displayed XRD patterns characteristic of a polycrystalline In_2_S_3_, with relatively similar peak intensities in the β‐phase In_2_S_3_ tetragonal structure. This disparity can be attributed to the conductive properties and rough surface morphology of the In_2_O_3_ NRs compared to the insulating and smooth surface of the SiO_2_ wafer. The rough In_2_O_3_ surface hindered the uniform growth of In_2_S_3_, resulting in random nucleation of In_2_S_3_ seeds, which subsequently grew along various crystalline facets.^[^ ^39^ ^]^

Raman spectroscopy was conducted to analyze the atomic vibration modes as a function of In_2_S_3_ nanocrystals on In_2_O_3_ NRs, with a particular focus on the differences between bare In_2_O_3_ NRs and 3‐ISO samples (Figure S4, Supporting information). Before In_2_S_3_ deposition, the characteristic Raman modes of In_2_O_3_, identified as E_1g_ (303 cm^−1^) and A_1g_ (495 cm^−1^), were observed, confirming the highly crystalline structure of the In_2_O_3_ NRs.^[^ ^40^ ^]^ For the 3‐ISO sample, additional peaks corresponding to In_2_S_3_, specifically B_2g_ (245 cm^−1^) and E_g_ (308 cm^−1^), appeared, while the In_2_O_3_ peaks diminished.^[^ ^41^ ^]^ As the In_2_S_3_ formed a continuous layer over the In_2_O_3_ surface, Raman peaks from both materials coexisted, although the In_2_O_3_ signals persisted. With increased growth time, the In_2_S_3_ peaks became dominant, and the In_2_O_3_ Raman modes were nearly suppressed, indicating full surface coverage by In_2_S_3_. X‐ray photoelectron spectroscopy (XPS) analysis was performed to investigate the surface compositional changes during the deposition process, with detailed explanations provided in Figure S5 (Supporting information).

Figure 1d presents high‐resolution transmission electron microscopy (HR‐TEM) images with fast Fourier transform (FFT) analysis for the 3‐ISO sample, enabling structural characterization of the ISO heterostructures. The cubic In_2_O_3_ phase was identified by a lattice fringe spacing of 0.29 nm, corresponding to the (222) plane with the standard reference (JCPDS‐066‐0187). The tetragonal In_2_S_3_ phase was confirmed with lattice fringe spacings of 0.50 and 0.74 nm, which correspond to the (015) and (101) planes (JCPDS‐025‐0390). These results indicate that both In_2_O_3_ and In_2_S_3_ were synthesized with high crystallinity. Energy‐dispersive X‐ray spectroscopy (EDS) analysis for the 3‐ISO is shown in Figure S6 (Supporting Information), demonstrating that the In_2_S_3_ was uniformly distributed across the surface and penetrated lower regions of the In_2_O_3_ nanorods.

### 2.2 Gas Sensing Properties and Mechanism of In_2_S_3_‐In_2_O_3_

**Figure 2:**
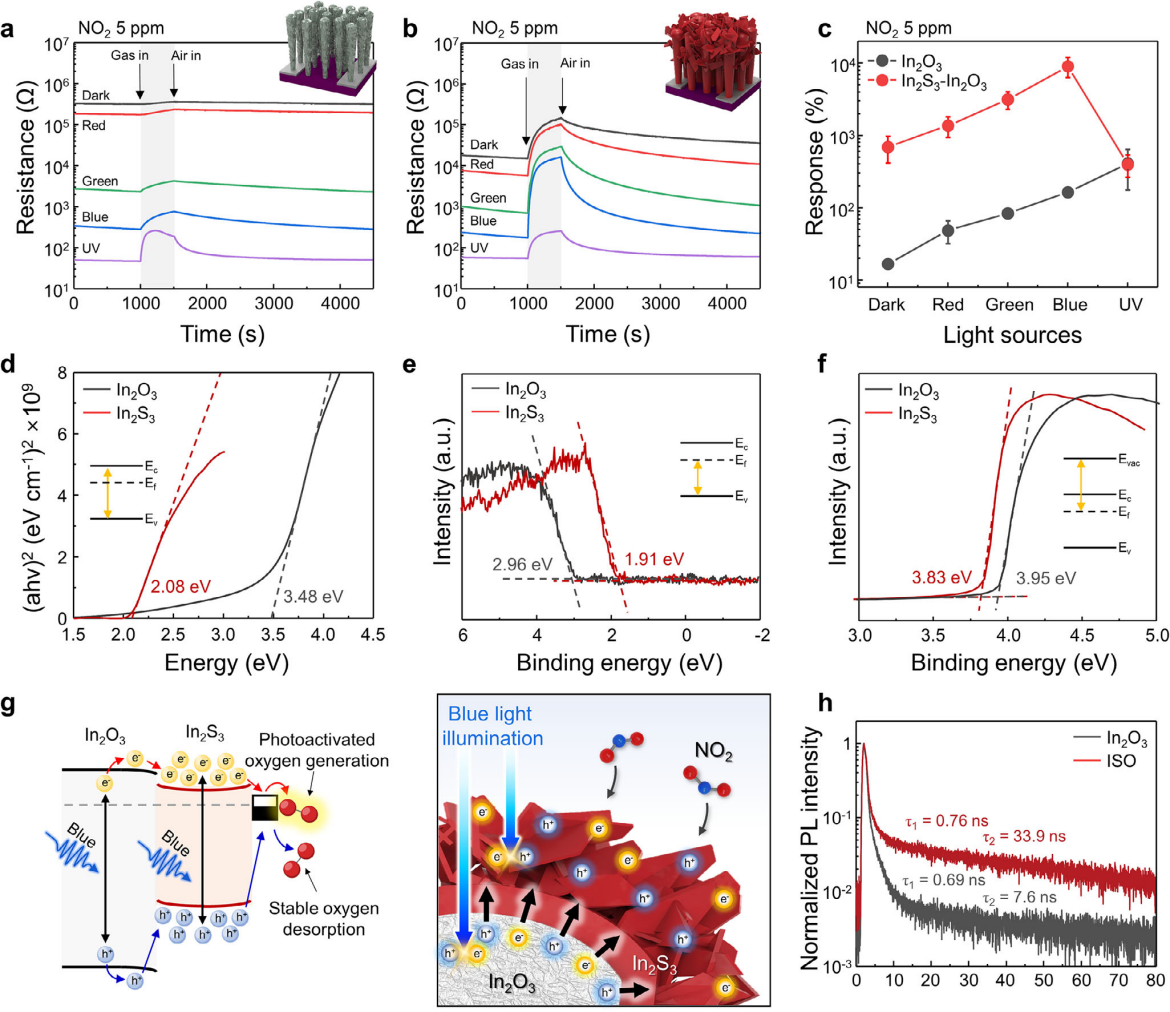
Dynamic R‐t curves of a) In_2_O_3_ nanorods and b) 3‐ISO to 5 ppm of NO_2_ under various light sources (Red: 620–625 nm, Green: 510–515 nm, Blue: 450–455 nm, and UV light: 395‐400 nm). c) Response plots of In_2_O_3_ nanorods and 3‐ISO–5 ppm of NO_2_ under various light sources. d) Tauc plots, e) secondary electron emission spectra, and f) valence band spectra of In_2_O_3_ nanorods and In_2_S_3_ film. g) Schematic illustration of the mechanism of NO_2_ sensing enhancement for ISO under blue light illumination. h) TRPL decay spectra of In_2_O_3_ nanorods and 3‐ISO.

The gas sensor measurement system under light illumination was established using commercial LED bulbs (Figure S7, Supporting information). The 3‐ISO was selected as the optimized structure among the ISO samples based on the comparative sensing properties, as shown in Figure S8 (Supporting Information). Thus, the gas‐sensing properties of bare In_2_O_3_ NRs and 3‐ISO samples were evaluated under both dark conditions and light illumination using red, green, blue, and UV light at 5 ppm of NO_2_, which corresponds to the Occupational Safety and Health Administration (OSHA) ceiling permissible exposure limit (**Figure** **2**a–c). Upon exposure to 5 ppm of NO_2_, the resistance of both sensors significantly increased, consistent with the behavior of conventional *n*‐type semiconductor gas sensors.^[^ ^42^ ^]^ Under light illumination, both bare In_2_O_3_ NRs and 3‐ISO samples exhibited enhanced responses to NO_2_, with variations depending on the illumination wavelength compared to dark conditions. For bare In_2_O_3_ NRs, the gas response to 5 ppm NO_2_ increased as the illumination wavelength decreased, peaking under UV light with a response of 411%. In contrast, the 3‐ISO sample demonstrated the highest response under blue light, achieving a remarkable 9202%, 56 times greater than bare In_2_O_3_ NRs. Moreover, both sensor types exhibited shorter response times as the illumination wavelength decreased, with the 3‐ISO sensor demonstrating faster responses across all wavelengths compared to bare In_2_O_3_ NRs. Notably, the 3‐ISO device achieved the fastest recovery under blue light, with a recovery time 10 times shorter than that of bare In_2_O_3_. These results highlight the superior NO_2_ response and recovery performance of the ISO‐based sensor, particularly under blue light illumination.

To understand these trends, the band structure of the ISO heterostructure was analyzed using UV–vis absorption spectroscopy, secondary electron emission spectroscopy, and ultraviolet photoelectron spectroscopy (UPS), as shown in Figure 2d–f. From Tauc plots derived from UV–vis spectra (Figure 2d), the optical bandgap of In_2_S_3_ was determined to be 2.08 eV, corresponding to green light, while that of In_2_O_3_ was 3.48 eV, corresponding to UV light (Figure S9, Supporting Information). The secondary electron emission spectra (Figure 2e) revealed that the energy differences between the valence band and the Fermi level for In_2_S_3_ and In_2_O_3_ were 1.91 and 2.96 eV, respectively. UPS analysis determined the work functions of In_2_S_3_ and In_2_O_3_ as 3.83 and 3.95 eV, respectively (Figure 2f). These data indicate that the combination of In_2_S_3_ and In_2_O_3_ forms a type‐I heterojunction (Figure S10, Supporting Information).

Under green light illumination, the photon energy is close to the bandgap energy of In_2_S_3_ but remains below the bandgap of In_2_O_3_. As a result, only In_2_S_3_ is photoactivated, which limits overall photoactivation in the ISO structure. Under UV illumination, the photon energy lies above the band gaps of both materials. In_2_O_3_ is efficiently excited, whereas in In_2_S_3_, the excess photon energy generates high‐energy e‐h pairs that recombine rapidly, which limits the contribution to photoactivated gas reactions.^[^ ^34^, ^43^ ^]^ Blue light lies between the band gaps of In_2_S_3_ and In_2_O_3_. At this wavelength, both In_2_S_3_ and In_2_O_3_ absorb photons and generate e‐h pairs. In the type‐I heterojunction, the photogenerated electrons (e^−^_(_ *_hv_*_)_) and holes (h^+^_(_ *_hv_*_)_) in In_2_O_3_ are transferred to In_2_S_3_, while the electron‐hole pairs generated in In_2_S_3_ remain on its surface (Figure 2g). This process leads to the accumulation of charge carriers on the In_2_S_3_ surface, enhancing its surface reactivity. The h^+^_(_ *_hv_*_)_ reacts with absorbed oxygen species on the In_2_S_3_ surface, desorbing them and generating active sites (Equation 2). Simultaneously, the e^−^_(_ *_hv_*_)_ reacts with ambient oxygen to produce photoactivated oxygen (O_2_^−^_(_ *_hv_*_)_) species (Equation 3). These activated oxygen species exhibit high reactivity with NO_2_, forming NO_2_^−^ through reactions with oxygen species, direct electron transfer, or further oxidation to surface nitrates (NO_3_^−^) (Equations (4), (5), (6)).^[^ ^11^ ^]^ During recovery, the h^+^_(_ *_hv_*_)_ recombine with adsorbed NO_2_^−^, leading to its desorption (Equation 7), or decomposition of NO_3_^−^ (Equation 8).^[^ ^44^, ^45^, ^46^ ^]^

(2) $$O_{2(\mathrm{ad})}^{-}+h_{\left(\mathrm{hv}\right)}^{+}\to O_{2(g)}$$

(3) $$O_{2}\left(g\right)+{e^{-}}_{\left(\mathrm{hv}\right)}\to O_{2\;\left(\mathrm{hv}\right)}^{-}$$

(4) $$NO_{2(g)}+O_{2\left(\mathrm{hv}\right)}^{-}\to {\mathrm{NO}}_{2\left(\mathrm{ad}\right)}^{-}+O_{2(g)}$$

(5) $$2NO_{2(g)}+O_{2\left(\mathrm{hv}\right)}^{-}\to {2\mathrm{NO}}_{3(\mathrm{ad})}^{-}$$

(6) $$NO_{2(g)}+e_{\left(\mathrm{hv}\right)}^{-}\to {\mathrm{NO}}_{2(\mathrm{ad})}^{-}$$

(7) $${\mathrm{NO}}_{2(\mathrm{ad})}^{-}+h^{+}\to NO_{2(g)}$$

(8) $${\mathrm{NO}}_{3(\mathrm{ad})}^{-}+hv\to NO_{\left(g\right)}+O_{2(g)}$$

Based on the proposed chain reactions, the e^−^_(_ *_hv_*_)_ and h^+^_(_ *_hv_*_)_ pairs on the ISO surfaces are critical factors for gas detection. Consequently, the type‐I ISO heterojunction exhibited excellent NO_2_ detection properties under blue light illumination. Furthermore, time‐resolved photoluminescence (TRPL) analysis was performed on the bare In_2_O_3_ NRs and ISO samples using a 450 nm laser corresponding to blue light (Figure 2h; Figure S11, Supporting Information) to investigate the charge carrier kinetics. The fast decay (*τ*_1_) reflects defect‐related electron trapping, while the slow decay (*τ*_2_) represents electron‐hole recombination.^[^ ^47^ ^]^ The fast decay time was similar for both ISO samples (0.76 ns) and bare In_2_O_3_ NRs (0.69 ns). However, the *τ*_2_ was significantly longer for the ISO sample (33.9 ns) than bare In_2_O_3_ NRs (7.6 ns). These results suggest that the 3‐ISO heterostructure effectively suppresses the recombination of photogenerated electron‐hole pairs by transferring charge carriers to the In_2_S_3_ surface, where they react with adsorbed oxygen species and target gases, thereby enabling a high‐performance NO_2_ gas response.

**Figure 3:**
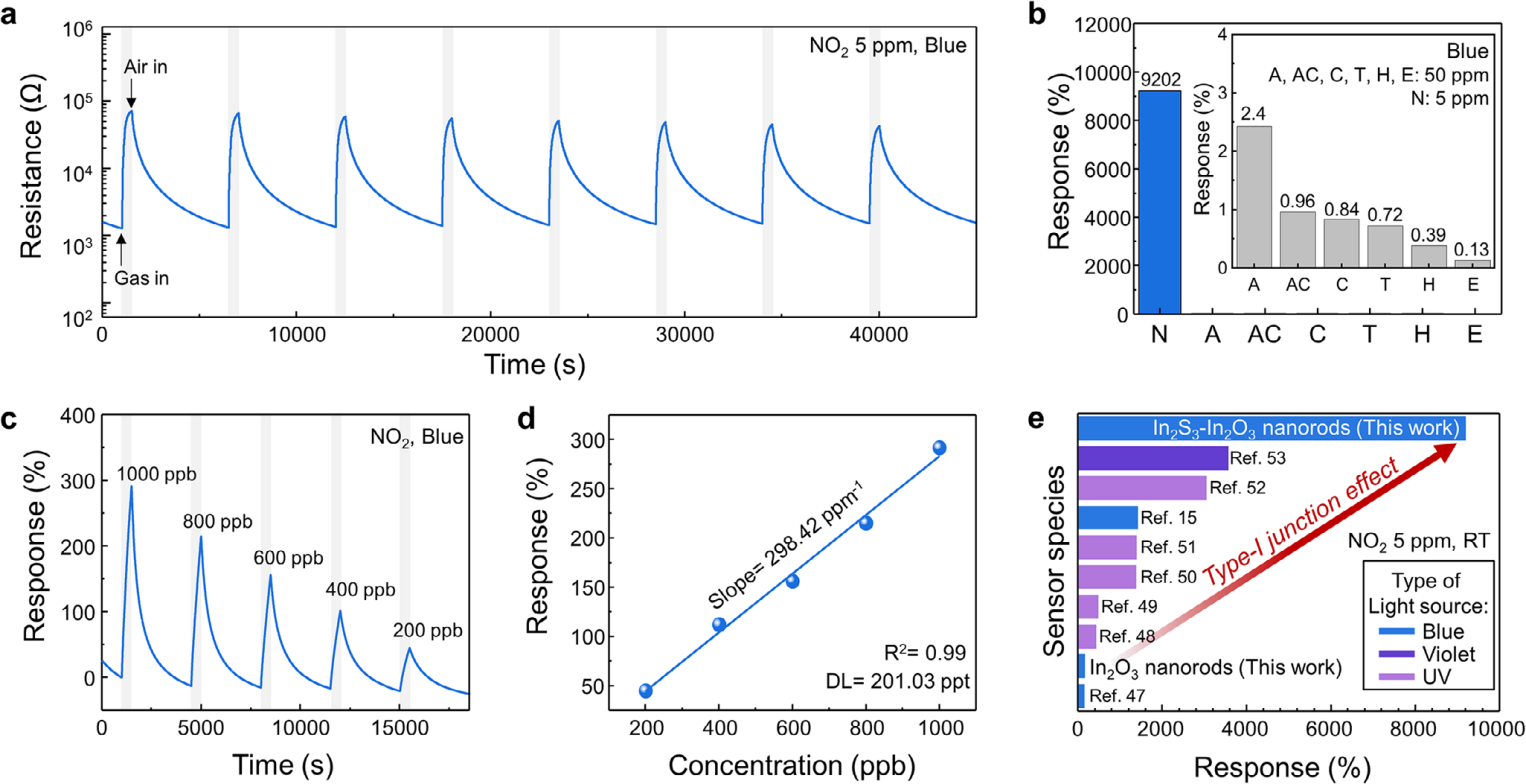
a) Response curves of 3‐ISO to eight pulses of 5 ppm of NO_2_ under blue light illumination. b) Selectivity of 3‐ISO to various gases under blue light illumination (N: NO_2_, A: NH_3_, AC: CH_3_COCH_3_, C: CO, T: C_7_H_8_, E: C_2_H_5_OH, H: H_2_). c) Response curves and d) response calibration of 3‐ISO to 200–1000 ppb of NO_2_. e) Comparison of the response to the state‐of‐the‐art of light‐activated NO_2_ gas sensors.

To further assess the suitability of the ISO structure for NO_2_ sensing, additional measurements were conducted on 3‐ISO under blue light illumination. The reliability of 3‐ISO was confirmed through eight repetitive cycles of 5 ppm NO_2_ exposure (**Figure** **3**a). The sensor demonstrated consistent responses and fully recovered to its baseline after each cycle, confirming its stability and repeatability. Furthermore, the NO_2_ sensing properties of 3‐ISO under blue light illumination show a higher response than its performance at elevated temperatures of 100, 150, and 200 °C (Figure S12, Supporting Information). These results indicate that light activation is more effective than thermal activation in enhancing the gas‐sensing performance of the ISO heterostructure. The selectivity of 3‐ISO was evaluated by exposing it to 50 ppm of various interfering gases, including CH_3_COCH_3_, NH_3_, C_2_H_5_OH, H_2_, CO, and C_7_H_8_ (Figure 3b; Figure S13, Supporting Information). Remarkably, the response to 5 ppm of NO_2_ was 3834 times greater than the second‐highest response observed for 50 ppm NH_3_. This outstanding selectivity demonstrates the ability of the sensors to distinguish NO_2_ from other gases effectively. 3‐ISO was exposed to NO_2_ concentrations of 200, 400, 600, 800, and 1000 ppb to evaluate sensitivity under blue light illumination (Figure 3c,d). The gas response exhibited a strong linear correlation with NO_2_ concentration, with an *R* ^2^ of 0.99 and a 298.42 ppm^−1^ slope. The limit of detection (LOD), defined as the concentration at which the signal‐to‐noise ratio of 3, was calculated to be 201.03 ppt, highlighting the excellent sensitivity of the 3‐ISO‐based gas sensor.

The response of 3‐ISO was also tested under various humidity levels, including dry air, 20% RH (relative humidity), 50% RH, and 80% RH, to 2 ppm of NO_2_ (Figure S14, Supporting Information). The baseline resistance of 3‐ISO decreased with increasing RH due to the hopping charge transport mechanism involving OH^−^ ions, as described in Equation (7).^[^ ^48^, ^49^ ^]^

(9) $$H_{2}O_{\left(g\right)}\to H_{\left(\mathrm{ad}\right)}^{+}+{\mathrm{OH}}_{\left(\mathrm{ad}\right)}^{-}$$

The response to NO_2_ increased at higher RH levels, as pre‐chemisorbed H^+^ ions facilitated NO_2_ adsorption.^[^ ^50^ ^]^ These results emphasize the excellent NO_2_ detection performance of 3‐ISO even in humid environments. The long‐term stability of 3‐ISO was demonstrated by its ability to maintain a stable baseline resistance and reliable NO_2_ response observed after 30 and 294 days (Figure S15, Supporting Information). To highlight the superior gas‐sensing properties of 3‐ISO, its performance was compared with the state‐of‐the‐art light‐activated gas sensors, as summarized in Figure 3e and Table S2 (Supporting Information).^[^ ^15^, ^51^, ^52^, ^53^, ^54^, ^55^, ^56^, ^57^ ^]^ While bare In_2_O_3_ NRs exhibited lower response than reported references, the 3‐ISO heterostructure achieved the highest response, even under visible light activation. These findings confirm that the 3‐ISO type‐I heterojunction significantly enhances NO_2_ detection by optimizing the light activation effect.

### 2.3 Noble Metal‐Decorated 2 × 2 Array

To broaden the application of the ISO type‐I junction, we fabricated a 2 × 2 sensor array comprising four distinct sensor types via the noble‐metal decoration of 3‐ISO. Pd, Pt, and Au were selected because their distinct catalytic effects modulate selectivity toward different analytes, and their use in gas sensor arrays is well established in our previous works.^[^ ^8^, ^36^ ^]^ Each noble metal was deposited onto 3‐ISO via a solution‐based process (Figure S16a, Supporting Information). The 3‐ISO samples were immersed in an aqueous precursor solution containing noble metal salts (K_2_PdCl_4_, K_2_PtCl_4_, and KAuCl_4_) and maintained at 70 °C. Under these conditions, metal chloride ions undergo redox reactions from the variable valence In sites on the In_2_S_3_ surface with uniform nucleation and growth of metal nanoparticles (NPs) (Figure S16b, Supporting Information).^[^ ^58^ ^]^ The concentration of each noble metal precursor was selected by optimizing the sensing performance (Figure S17, Supporting Information). Each noble metal‐decorated 3‐ISO is denoted accordingly: Pd@3‐ISO for Pd‐decorated, Pt@3‐ISO for Pt‐decorated, and Au@3‐ISO for Au‐decorated samples.

**Figure 4:**
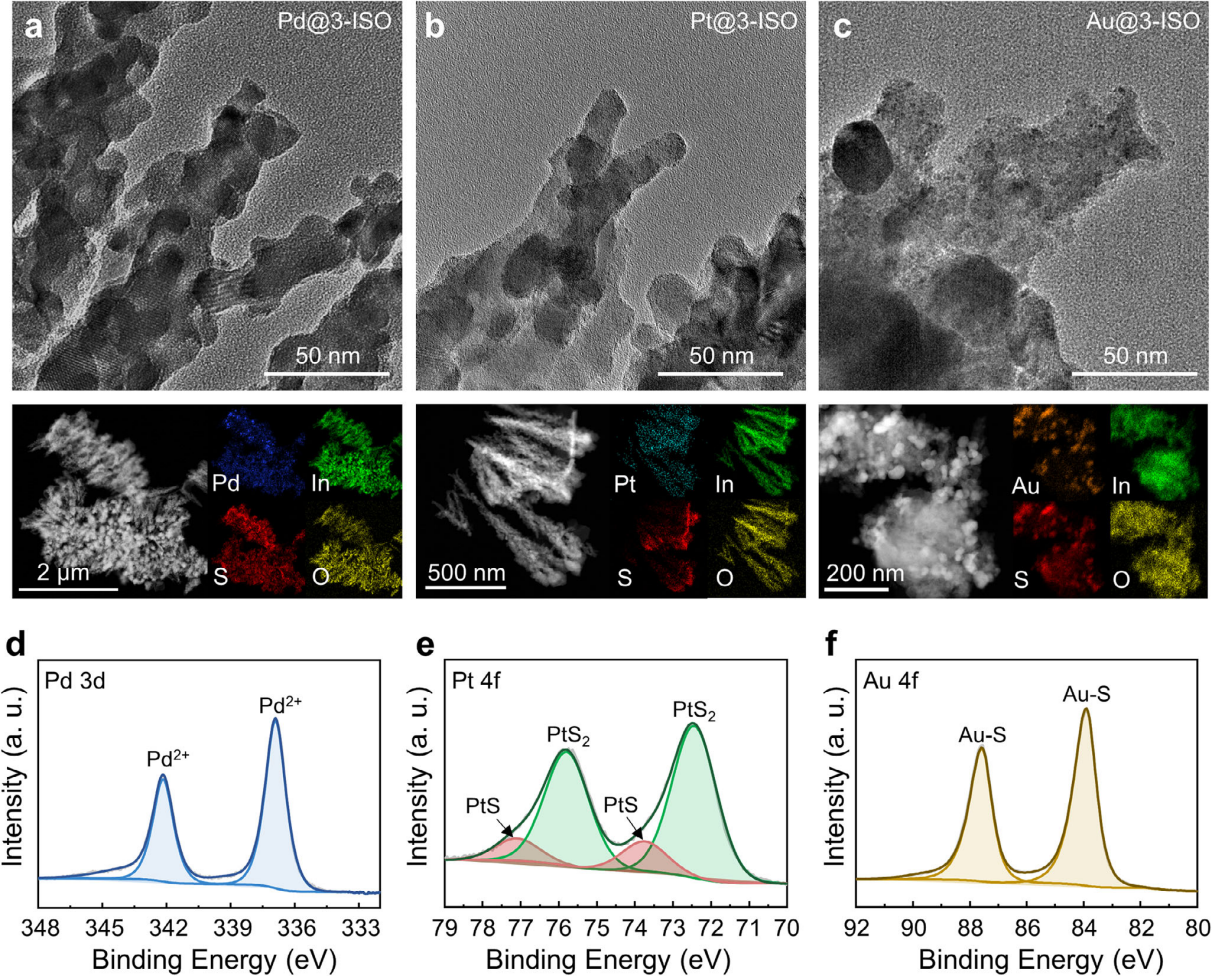
TEM images and corresponding EDS mappings of a) Pd@3‐ISO, b) Pt@3‐ISO, and c) Au@3‐ISO, respectively. XPS spectra of d) Pd 3d for Pd@3‐ISO, e) Pt 4f for Pt@3‐ISO, f) Au 4f for Au@3‐ISO.

SEM images of each noble‐metal‐decorated 3‐ISO show preserved porosity and retention of the nanorod morphology (Figure S18, Supporting Information). In the XRD patterns, noble metal peaks are difficult to resolve because of the low amount, whereas the distinct In_2_O_3_ and In_2_S_3_ peaks indicated that the ISO structure is maintained (Figure S19, Supporting Information). TEM and EDS analysis were conducted to provide structural and compositional confirmation of the noble metal decoration (**Figure** **4**a–c). Pd NPs in Pd@3‐ISO appeared blurred in TEM images due to the contrast limitations, but EDS mapping indicated a uniform Pd distribution across the entire structure. Pt@3‐ISO and Au@3‐ISO samples exhibited uniformly distributed small metal NPs in TEM and EDS analysis. These results support that the solution‐based deposition ensures uniform noble metal decoration, even within deeper regions of the 3‐ISO nanostructures. XPS analyses further validated the noble metal decoration onto ISO heterostructures. The XPS survey spectra and corresponding deconvoluted spectra of In and O are presented in Figure S20 (Supporting Information) with supplementary explanations. In the deconvoluted spectra of noble metals, each ISO shows the clear metal peaks in Pd 3d, Pt 4f, and Au 4f (Figure 4d–f). The Pd 3d spectra showed peaks at 336.9 eV associated with PdS, indicating robust interaction and stable metal incorporation.^[^ ^59^, ^60^ ^]^ The Pt 4f spectra exhibited peaks corresponding to PtS_2_ at 72.4 eV and PtS at 73.6 eV, confirming strong metal‐sulfur interactions.^[^ ^61^ ^]^ Au 4f spectra displayed distinct peaks at 83.9 eV, corresponding to Au─S bonding in a stable state with effective decoration.^[^ ^62^ ^]^ This suggested that the noble metals are chemically coupled to the surface of In_2_S_3_. Consequently, the XPS results confirm the uniform decoration of Pt, Pd, and Au onto ISO nanostructures.

**Figure 5:**
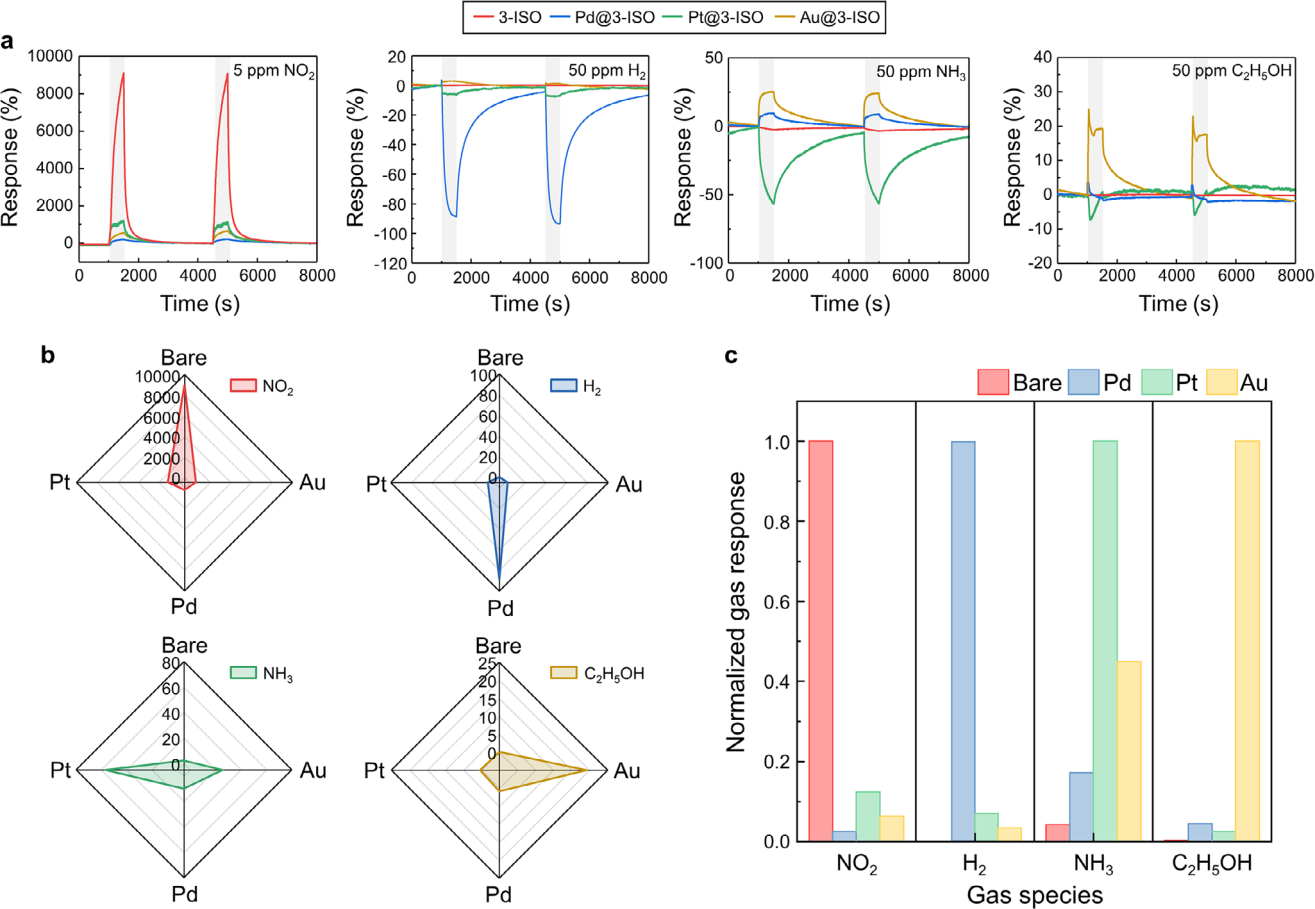
a) Response curves and b) radar plots of bare 3‐ISO, Pd@3‐ISO, Pt@3‐ISO, and Au@3‐ISO to 5 ppm of NO_2_ and 50 ppm of H_2_, NH_3_, and C_2_H_5_OH. c) Normalized response plots of bare 3‐ISO, Pd@3‐ISO, Pt@3‐ISO, and Au@3‐ISO to NO_2_, H_2_, NH_3_, and C_2_H_5_OH.

The gas sensing performance of the ISO‐based 2 × 2 sensor array was evaluated for various gases, including 5 ppm of NO_2_, and 50 ppm of reducing gases with NH_3_, C_2_H_5_OH, and H_2_ to calibrate and compare the selectivity across each sensor. The ISO‐based sensors were tested under dark, red, green, and blue light illumination, and blue light exhibited the most favorable sensing properties (Figure S21, Supporting Information). Accordingly, the additional measurements of the sensor array were conducted under blue light illumination (**Figure** **5**a,b). For NO_2_ detection, the bare 3‐ISO sample showed the highest response, attributed to the direct adsorption of NO_2_ on the active sites of the ISO surface. Noble metal decoration, however, reduced the number of available adsorption sites, resulting in a decreased response to NO_2_. For reducing gases such as NH_3_, H_2_, and C_2_H_5_OH, the sensing mechanism involved adsorption onto the 3‐ISO surface, followed by a reaction with photoactivated oxygen species, described by the following equations:

(10) $$2\;NH_{3(g)}+3\;O_{2(\mathrm{hv})}^{-}\to 3\;H_{2}O_{\left(g\right)}+N_{2(g)}+2\;e^{-}$$

(11) $$H_{2(g)}+O_{2(\mathrm{hv})}^{-}\to 2\;H_{2}O+2\;e^{-}$$

(12) $$C_{2}H_{5}OH_{\left(g\right)}+{3\;O}_{2(\mathrm{hv})\;}^{-}\to 2\;CO_{2}+3\;H_{2}O+4e^{-}$$

The energy required for these reactions is insufficient at room temperature, leading to negligible responses from bare 3‐ISO. However, noble metal particles significantly lower the activation energy for these reactions, enhancing sensor performance. For H_2_ detection, the Pd@3‐ISO exhibited the highest response, which can be attributed to the ability of Pd to dissociate H_2_ molecules into H atoms through the formation of Pd‐H_x_ phase:^[^ ^58^, ^63^, ^64^ ^]^

(13) $$H_{2(g)}\to 2H$$

(14) $$4H+O_{2(\mathrm{hv})}^{-}\to 2\;H_{2}O_{\left(g\right)}+e^{-}$$

These H atoms readily react with photoactivated oxygen species, thereby enhancing the gas response. In the case of NH_3_, the Pt@3‐ISO sensor exhibited the highest response. This enhancement is attributed to the catalytic role of Pt, which facilitates the dissociation of O_2_^−^_(_ *_hv_*_)_ into more reactive O^−^_(_ *_hv_*_)_, thus lowering the activation energy required for NH_3_ oxidation.^[^ ^65^, ^66^ ^]^ Moreover, Pt provides an optimal interaction strength toward nitrogen‐containing intermediates during NH_3_ oxidation.^[^ ^67^, ^68^ ^]^ In catalytic NH_3_ sensing, the reaction proceeds through successive dehydrogenation steps involving adsorbed *NH_3_, *NH_2_, and *NH species. If the adsorption is too weak, NH_3_ molecules desorb before oxidation occurs, whereas excessively strong adsorption leads to surface poisoning by stable *N intermediates, which hinders further reactions. The moderate binding ability of Pt maintains a balanced adsorption‐oxidation cycle, allowing effective oxidation of NH_3_. This balanced interaction also facilitates interfacial electron exchange between Pt and the ISO substrate, accelerating surface reaction kinetics and leading to superior catalytic activity for NH_3_ detection. For C_2_H_5_OH detection, the Au@3‐ISO exhibited the highest response, which was characterized by an increase in resistance, opposite to the conventional behavior of *n*‐type gas sensors.^[^ ^42^ ^]^ Although Au NPs exhibit strong C_2_H_5_OH adsorption, the activation energy for C_2_H_5_OH oxidation is insufficient, leading to suppression of space charge transfer.^[^ ^69^ ^]^ Instead, the adsorbed C_2_H_5_OH molecules disrupt the electron transport in 3‐ISO, enhancing electron scattering and thereby increasing resistance.^[^ ^70^, ^71^ ^]^ To validate this mechanism, Au@3‐ISO was measured at elevated temperatures ranging from 100 to 200 ^°^C (Figure S22e,f, Supporting Information). At higher temperatures, space charge transfer dominates over electron scattering, resulting in a decrease in resistance, which is consistent with previous reports.^[^ ^70^ ^]^ UV–vis spectroscopy confirmed the localized surface plasmon resonance (LSPR) of the Au NPs in Au@3‐ISO (Figure S23, Supporting Information). Upon light illumination with blue light, the LSPR effect generates additional e‐h pairs, increasing the free carrier concentration and enhancing electron scattering upon interaction with gas molecules. This plasmon‐induced electronic modulation contributes to the unique resistance increase observed under C_2_H_5_OH exposure, thereby allowing the Au@3‐ISO sensor to selectively discriminate C_2_H_5_OH from other gases.^[^ ^16^ ^]^

Compared with high‐temperature operation, the blue light‐activated ISO array exhibits room‐temperature responses that approach those at elevated temperature (Figure S21, Supporting Information). The 2 × 2 array also showed repetitive detection over eight pulses of gas reactions and a linear response with ppb‐level detection limits (Figures S24 and S25, Supporting Information). Figure S26 (Supporting Information) presents the sensing properties of a 2 × 2 array under various humid conditions. For gas mixtures of NO_2_, H_2_, C_2_H_5_OH, and NH_3_, the 2 × 2 array yields distinct response patterns (Figure S27, Supporting Information). These results demonstrate that noble metal particles impart distinct catalytic effects to the sensor, enabling selective detection of specific gases under blue light illumination.

The normalized response plots of the 2 × 2 sensor array exhibit distinct response tendencies with 3‐ISO responding to NO_2_, Pd@3‐ISO to H_2_, Pt@3‐ISO to NH_3_, and Au@3‐ISO to C_2_H_5_OH for Au@3‐ISO (Figure 5c). Compared to other noble‐metal decorated 2 × 2 gas sensor arrays reported in the previous works, such as SnO_2_ NPs activated by µ‐LED^[^ ^8^ ^]^ or MoS_2_ flakes operated at high temperatures,^[^ ^36^ ^]^ the ISO‐based sensor array achieves excellent selective gas sensing performance under commercial blue‐LED illumination by effectively maximizing light activation through the type‐I heterostructure (Figure S28, Supporting Information). With the expansion of the set of metal decorations, such as Ag, Ru, and Ir, the ISO‐based sensor array could further broaden the range of detectable gas species.

## 3 Conclusion

This study demonstrates the superior gas‐sensing capabilities of the ISO type‐I heterojunction, particularly under blue light illumination. The optimized 3‐ISO structure exhibited outstanding NO_2_ detection properties, including high sensitivity, excellent selectivity, rapid response and recovery times, and long‐term stability. The enhanced performance can be attributed to the efficient transfer of photogenerated charge carriers facilitated by the type‐I heterojunction. Furthermore, noble metal decoration on 3‐ISO expanded the application potential by enabling selective detection of reducing gases such as NH_3_, H_2_, and C_2_H_5_OH. Pd, Pt, and Au nanoparticles imparted unique catalytic effects, enhancing the response to specific gases and enabling effective gas discrimination within a 2 × 2 sensor array. These results highlight the capability of the ISO structure to overcome the limitations of conventional gas sensors, particularly the reliance on high‐temperature activation, by leveraging light activation to achieve exceptional performance. These findings indicate the potential for the next‐generation light‐activated gas sensors for diverse applications, including environmental monitoring, industrial safety, and healthcare.

## 4 Experimental Section

### Deposition of In_2_O_3_ Nanorods

The substrate for the gas sensor was fabricated with 5 µm IDEs. The electrodes on the IDEs were manufactured via e‐beam evaporation, deposing Pt (80 nm) and Ti (20 nm) layers onto a SiO_2_ wet‐oxidized substrate on a *p*‐type Si wafer. In_2_O_3_ NRs were deposited on the IDEs using GLAD in an e‐beam evaporator with In_2_O_3_ sources (99.99%, Taewon Scientific Co.). The chamber vacuum was maintained at a pressure of 2 × 10^−6^ Torr, with a deposition rate of 2 Å·s^−1^, a glancing angle of 80°, and a rotation speed of 80 rpm. To enhance the crystallinity of In_2_O_3_, the deposited In_2_O_3_ NRs were annealed in air at 550 °C for 2 h, with a ramping rate of 5 °C/min.

### Fabrication of In_2_S_3_‐In_2_O_3_ Heterostructures

Following the deposition of In_2_O_3_ NRs, an additional In_2_S_3_ layer was introduced onto the nanorods via a CVD process. The precursor materials used for this deposition were sulfur powder (99.99%, Sigma–Aldrich) and indium acetate (99.99%, Sigma–Aldrich), which were placed in separate quartz boats at designated locations within the heating zone of the CVD system. Specifically, indium acetate was positioned at the center of the heating zone, while sulfur and the In_2_O_3_ nanorod‐coated substrates were placed symmetrically at 25 cm upstream and downstream distances, respectively. The furnace was ramped to 850 °C during the reaction under a continuous flow of Ar (200 sccm) and H_2_ (20 sccm). A carrier gas mixture of H_2_ and Ar gases transported sulfur vapor through the chamber toward the indium acetate. The deposition process was carried out at this temperature for varying durations (1–10 min) to control the thickness and coverage of In_2_S_3_. The vaporized sulfur and indium acetate reacted in the heating zone, forming In_2_S_3_ vapor, which subsequently deposited onto the In_2_O_3_ NRs.

### Noble Metal Decoration on In_2_S_3_‐In_2_O_3_ Heterostructures

The metal precursors K_2_PdCl_4_ (98%, Sigma‐Aldrich), K_2_PtCl_4_ (98%, Sigma–Aldrich), and KAuCl_4_ (98%, Sigma–Aldrich) were diluted in distilled water at concentrations of 176, 3060, and 161 µm, respectively. The solutions were maintained at 70 °C, and the ISO sensors were immersed in the solution for 5 min. Following the completion of the reaction, ISO sensors were rinsed with dry air to remove the residue.

### Material Characterization

XRD patterns were measured using a SmartLab (Rigaku) diffractometer with Cu Kα radiation (λ = 1.5406 Å), operating at 45 kV and 200 mA. Raman spectra were acquired using a commercial confocal Raman microscope (WITec) equipped with a microscope and a 532 nm laser (resolution: 1 cm^−1^). SEM measurements were performed using a Hitachi S‐4800 at an accelerating voltage of 20 kV and a beam current of 20 µA. PL and TRPL measurements were conducted using a fluorescence spectrometer (FlouTime 300; PicoQuant) with an excitation laser of 405 nm. The TRPL decay curves were fitted using a biexponential decay model as shown in Equation (14):

(15) $$I\left(t\right)=A_{1}e^{-t/\tau_{1}}+A_{2}e^{-t/\tau_{2}}$$

where *I*(*t*) represents the intensity, *A*_1_ and *A*_2_ are amplitudes, and *τ*_1_ and *τ*_2_ correspond to the lifetimes of the fast and slow decay processes, respectively. The absorbance spectra were obtained using a UV‐vis spectrometer (V‐770; JASCO). Photoelectron spectroscopy (XPS and UPS) experiments were performed using a PHI5000 VersaProbe II system (ULVAC‐PHI, Japan) equipped with a monochromatic Al Kα source (1486.6 eV) and He(I) (21.2 eV) discharging lamp, respectively. The X‐ray beam spot size was set to 200 µm. The base pressure during XPS analysis was maintained below 3.5 × 10^−8^ Pa. The binding energy scale was calibrated following ISO 15472:2010. The sample surfaces were electrically connected to the sample holder using metal clips to minimize sample charging effects. High‐resolution core‐level spectra were acquired with a pass energy of 46.95 eV and an energy step size of 0.05 eV. All of the XPS peak fitting shown in this work was performed with CasaXPS software (version 2.3.26), where the fitting parameters were optimized to minimize the residual standard deviation (STD) between the measured and fitted XPS spectra. The valence band and work function measurements were carried out for UPS spectra using a pass energy of 0.58 eV and a step energy of 0.01 eV.^[^ ^72^ ^]^

### Gas Sensor Measurement

The fabricated sensor was placed inside a quartz tube to control the atmosphere and connected via Pt wires to an *I*‐*V* source meter (Keithley 2400) for resistance measurements. A commercial LED was inserted into the quartz tube and positioned at a distance of 2 cm above the sensors for light illumination, powered by the same electrical source from a power supply (Keithley 2231A‐30‐3). The wavelength and irradiance of the red, green, blue, and UV light sources were 620‐625 nm (11.9 mW·cm^−2^), 510‐515 nm (11.8 mW·cm^−2^), 450‐455 nm (12.0 mW·cm^−2^), and 395‐400 nm (12.1 mW·cm^−2^), respectively. A high‐temperature operation was conducted using a tube furnace (Thermo Scientific Lindberg/Blue M). The gases, including ambient air (21% O_2_ and 79% N_2_), 10 ppm of NO_2_, 100 ppm of H_2_, C_2_H_5_OH, NH_3_, CH_3_COCH_3_, and C_7_H_8_ with dry air balance, were purchased from Sinjin Gas Tech. The gas concentration was controlled using a mass flow controller by diluting with dry air, maintaining a total flow of 1000 sccm. For humid conditions, dry air was passed through a water bubbler, and the humid air was subsequently diluted with dry air to control the RH. The RH was calibrated using a humidity sensor at 25 °C.

The gas response with an increase in resistance (*R*_in_) and the gas response with a decrease in resistance (*R*_de_) were calculated using the following equations:

(16) $$R_{\mathrm{in}}=\left(R_{g}-R_{a}\right)/R_{a}\cdot 100\;\left(\%\right)$$

(17) $$R_{\mathrm{de}}=\left(R_{a}-R_{g}\right)/R_{g}\cdot 100\;\left(\%\right)$$

Here, *R*_g_ represents the resistance in the gas input state, and *R*_a_ denotes the resistance in the air input state. Response time is defined as the time required for the resistance to reach 90% of the maximum in the gas‐exposure state, and recovery time, defined as the time required to return to 90% of the baseline resistance in air.

The limit of detection (LOD) was calculated using the following equations:

(18) $$R_{x^{2}}=\sum {\left(\left(y_{i}-\bar{y}\right)\right.}^{2}$$

(19) $$\mathrm{rm}s_{\mathrm{noise}}=\sqrt{\frac{R_{x^{2}}}{N}}$$

(20) $$\mathrm{LOD}=3\frac{\mathrm{rm}s_{\mathrm{noise}}}{\mathrm{slope}}$$

Here, *y*_i_ represents the extracted data points from the dynamic curves of 3‐ISO, $\bar{y}$ is the average value of *y*_i_, and *N* is the number of extracted data points.

## Conflict of Interest

The authors declare no conflict of interest.

## Supporting information



Supporting Information

## Data Availability

The data that support the findings of this study are available from the corresponding author upon reasonable request.
