# Supplementary material for: Visible Light‐Driven Heterojunction Array Based on Type‐I In2S3/In2O3 for Selective Multi‐Gas Discrimination
Source: Small. 2025 Nov 18;22(1):e06056. doi: 10.1002/smll.202506056 (PMC12757980; doi:10.1002/smll.202506056)
Supplement: Supplementary file 1 — Supporting Information [file SMLL-22-e06056-s001.docx]

**Visible Light-Driven Heterojunction Array Based on Type-I In_2_S_3_/In_2_O_3_ for Selective Multi-Gas Discrimination**

*Gi Baek Nam, Jaekwon Ko, Seungwook Choi, Sungkyun Choi, Jin Wook Yang, Hee Ryeong Kwon, Yeong Jae Kim, Jihwan Kwon, Jongchul Jeon, Ansoon Kim, Young-Seok Shim, Seung-Wook Baek, In-Hyeok Park, Ho Won Jang^*^, Ki Chang Kwon^*^*

G. B. Nam, S. Choi, J. W. Yang, H. R. Kwon, Y. J. Kim, Prof. H. W. Jang

Department of Materials Science and Engineering

Research Institute of Advanced Materials

Seoul National University

Seoul 08826, Republic of Korea

J. Ko, J. Kwon, J. Jeon, S.-W. Baek, K. C. Kwon

Division of Chemical and Material Metrology

Korea Research Institute of Standards and Science

Daejeon 34133, Republic of Korea

J. Ko, S.-W. Baek, I.-H. Park

Graduate School of Analytical Science and Technology

Chungnam National University

Daejeon 34134, Republic of Korea

S. Choi, A. Kim

Strategic Technology Research Institute

Korea Research Institute of Standards and Science

Daejeon 34133, Republic of Korea

S. Choi, A. Kim, K. C. Kwon

Department of Applied Measurement Science

University of Science and Technology

Daejeon 34113, Republic of Korea

Y. S. Shim

School of Energy, Materials & Chemical Engineering

Korea University of Technology and Education

Cheonan, 31253 Republic of Korea

Prof. H. W. Jang

Advanced Institute of Convergence Technology

Seoul National University

Suwon 16229, Republic of Korea

Dr. K. C. Kwon

Department of Chemical Engineering

Chung-Ang University

Seoul 06974, Republic of Korea

*Corresponding author: H. W. Jang ([hwjang@snu.ac.kr](mailto:hwjang@snu.ac.kr)), K. C. Kwon (kichang.kwon@kriss.re.kr)


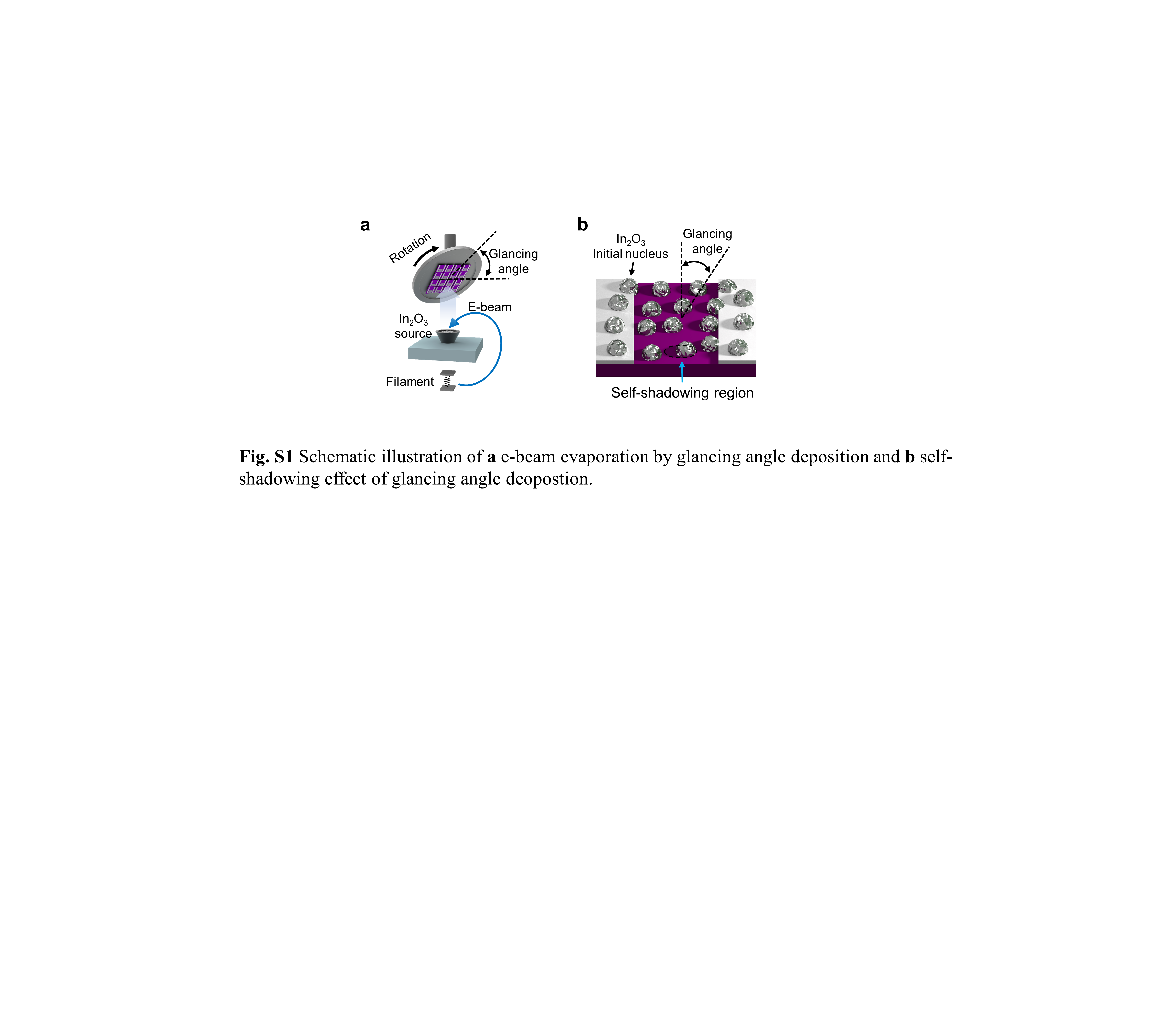


**Figure S1.** Schematic illustration of (a) e-beam evaporation by glancing angle deposition and (b) the self-shadowing effect of glancing angle deposition.


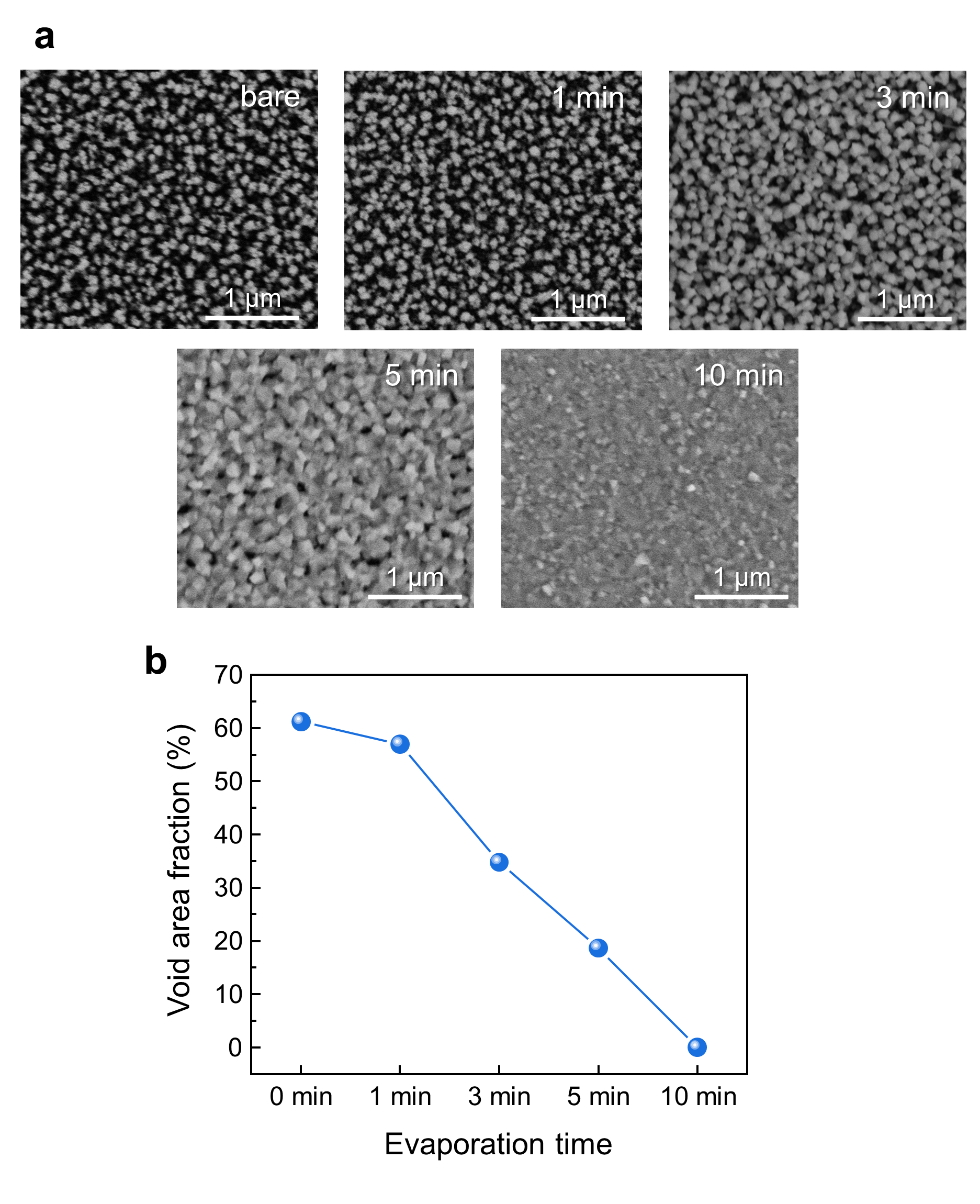


**Figure S2.** (a) SEM images and (b) void area fraction of ISO nanorods with different In_2_S_3_ evaporation times on In_2_O_3_ nanorods (0 min, 1 min, 3 min, 5 min, and 10 min).

Grayscale SEM images were converted to two-dimensional binary masks by thresholding (Otsu method) at fixed magnification and field of view. The void area fraction was calculated as follows.

$\phi_{A}\left( \% \right)=\frac{A_{void}}{A_{total}}*100$ (S1)


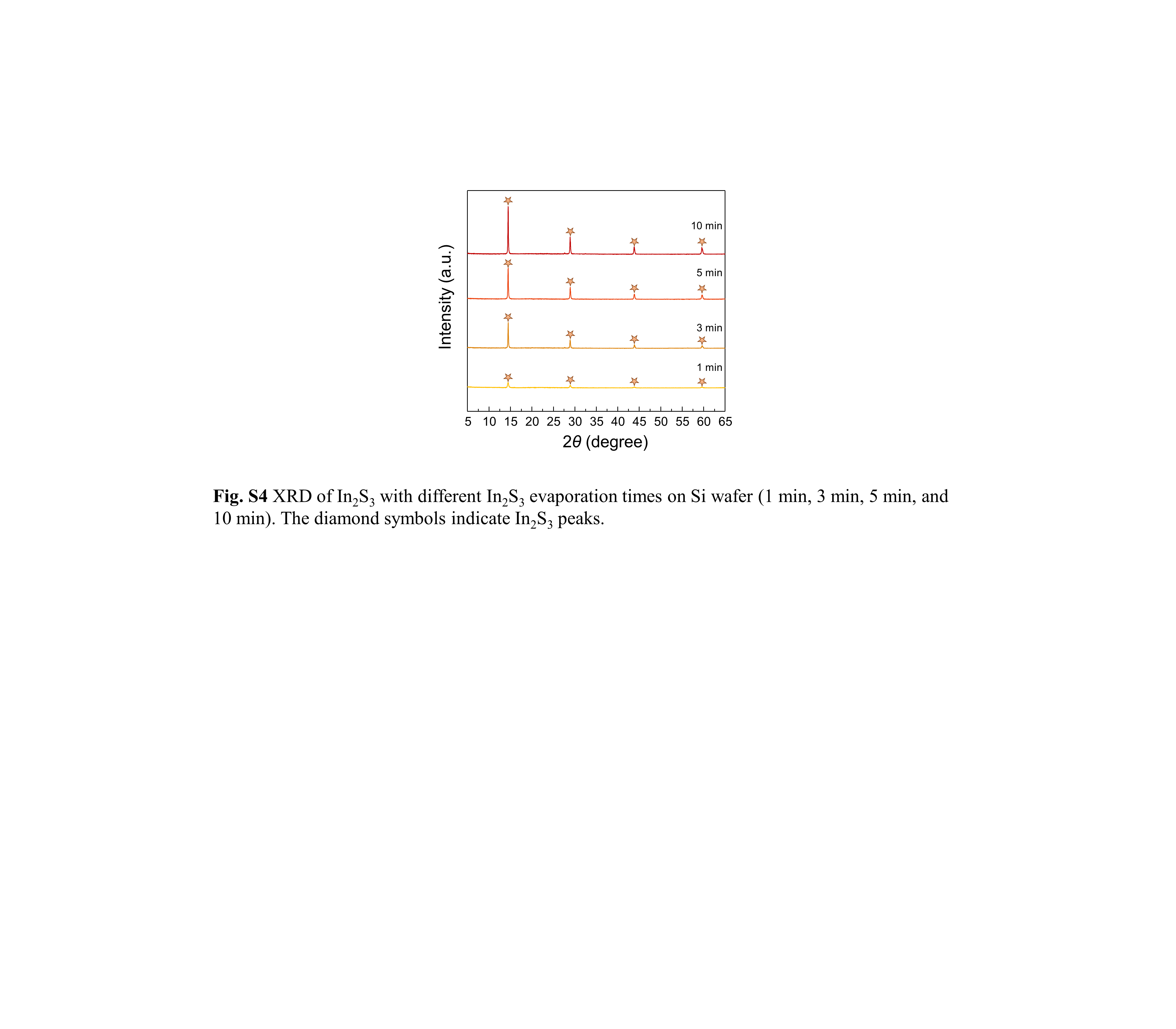


**Figure S3.** XRD of In_2_S_3_ with different In_2_S_3_ evaporation times on SiO_2_ wafer (1, 3, 5, and 10 min).


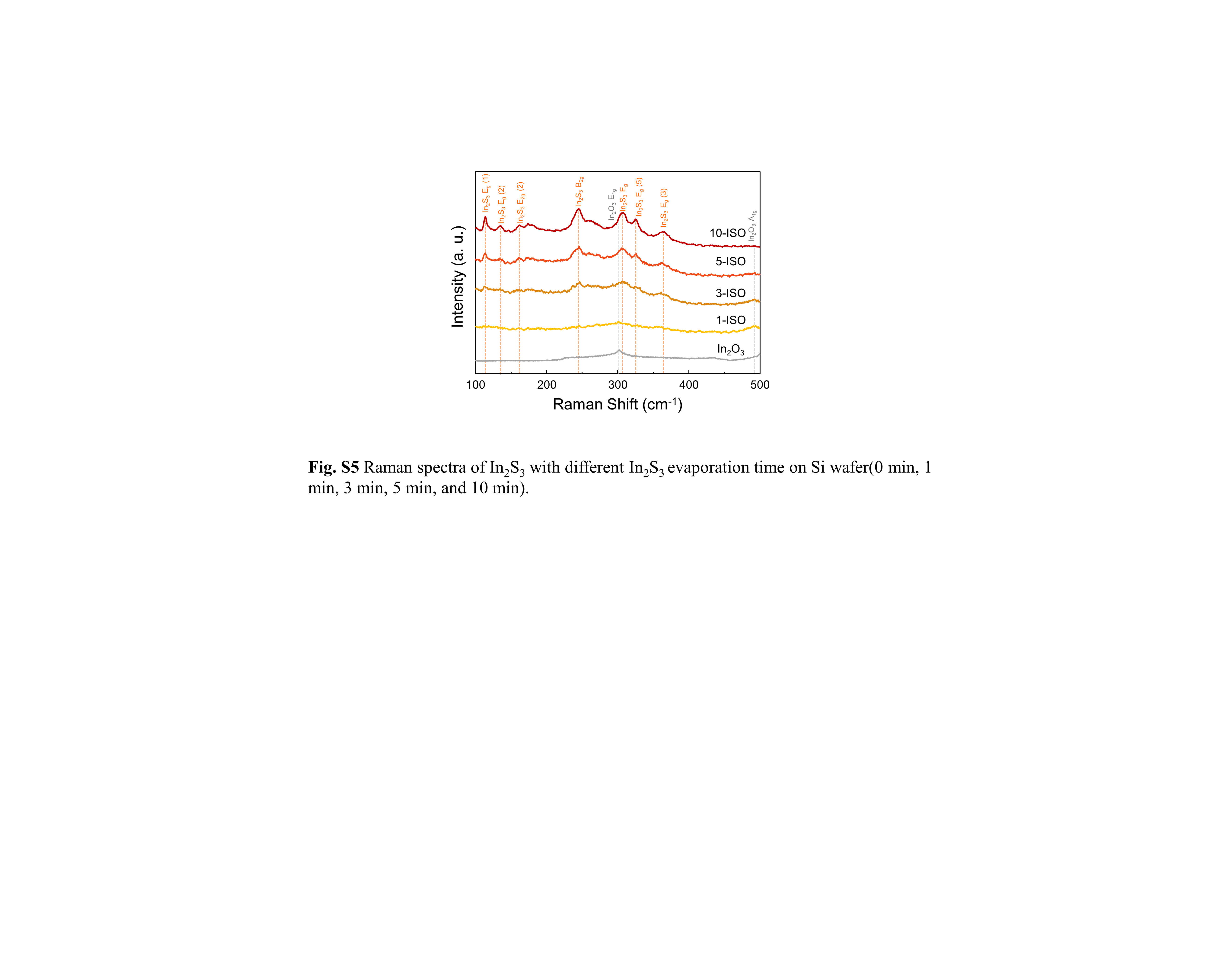


**Figure S4.** Raman spectra of In_2_S_3_ with different In_2_S_3_ growth times on a SiO_2_ wafer. The gray dotted line represents the peak corresponding to In_2_O_3_, and the red dotted line indicates the peak associated with In_2_S_3_.


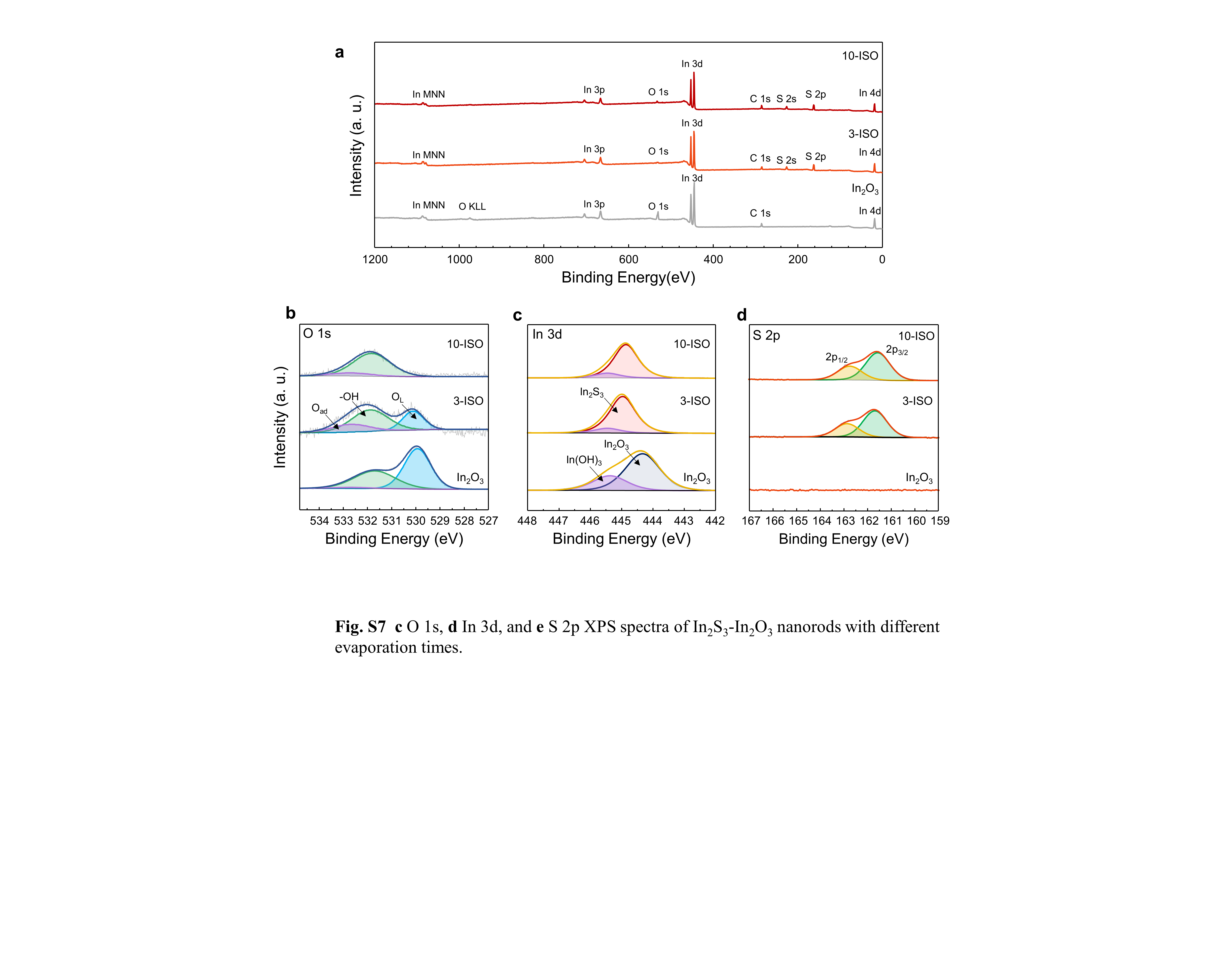


**Figure S5.** (a) XPS survey spectra of In_2_O_3_, 3-ISO, and 10-ISO. (b) O 1s, (c) In 3d, and (d) S 2p XPS spectra of In_2_O_3_, 3-ISO, and 10-ISO.

In XPS survey spectra, the In and O core level XPS spectra peaks were observed in In_2_O_3_, 3-ISO, and 10-ISO (Figure S5a). In contrast, the peak of the S 2*p* core level of XPS spectra was indicated in 3-ISO and 10-ISO. Deconvolution of the O 1*s* spectrum revealed a significant reduction in lattice oxygen (530.3 eV) after 3 minutes, accompanied by an increase in oxygen vacancies (531.8–531.9 eV) and surface-adsorbed oxygen (532.6 eV) (Figure S5b).^[R1-2]^ These changes indicate the onset of lattice rearrangement and sulfur species incorporation.

In the In 3*d*_5/2_ spectrum, both In_2_O_3_ (444.6 eV) and In_2_S_3_ (445.0 eV) peaks were detected at 3 minutes, showing the coexistence of the two materials (Figure S5c). Beyond 3 minutes, the In_2_O_3_ peak was no longer detectable, while In_2_S_3_ and In(OH)_3_ (445.3 eV) dominated, confirming that In_2_S_3_ had fully covered the surface.^[R3-4]^ Prolonged deposition resulted in minor shifts in peak intensity, indicating surface stabilization with slight reorganization. These results establish that 3 minutes marks the critical point where In_2_S_3_ deposition transitions from initiation to dominance, achieving optimal surface coverage while maintaining material stability.

In the S 2*p* spectrum (Figure S5d), peaks at 161.7 eV and 163.2–163.4 eV were observed, corresponding to In_2_S_3_ and mixed oxysulfide (InO_x_S_y_) phases.^[R5]^ At 3 minutes of deposition, the intensity of the In_2_S_3_ peak was significantly enhanced, indicating the effective incorporation of sulfur into the lattice and the formation of a robust In_2_S_3_ layer. The presence of minor oxysulfide peaks suggests the intermediate states during the gradual transition of surface composition to In_2_S_3_. Beyond 3 minutes, the In_2_S_3_ peak remained dominant, with no notable increase in intensity, while the oxysulfide peak slightly diminished, reflecting the stabilization of the In_2_S_3_ layer on the surface. These S 2*p* findings prove that 3 minutes is the critical deposition time for achieving optimal sulfur incorporation and surface coverage.

**
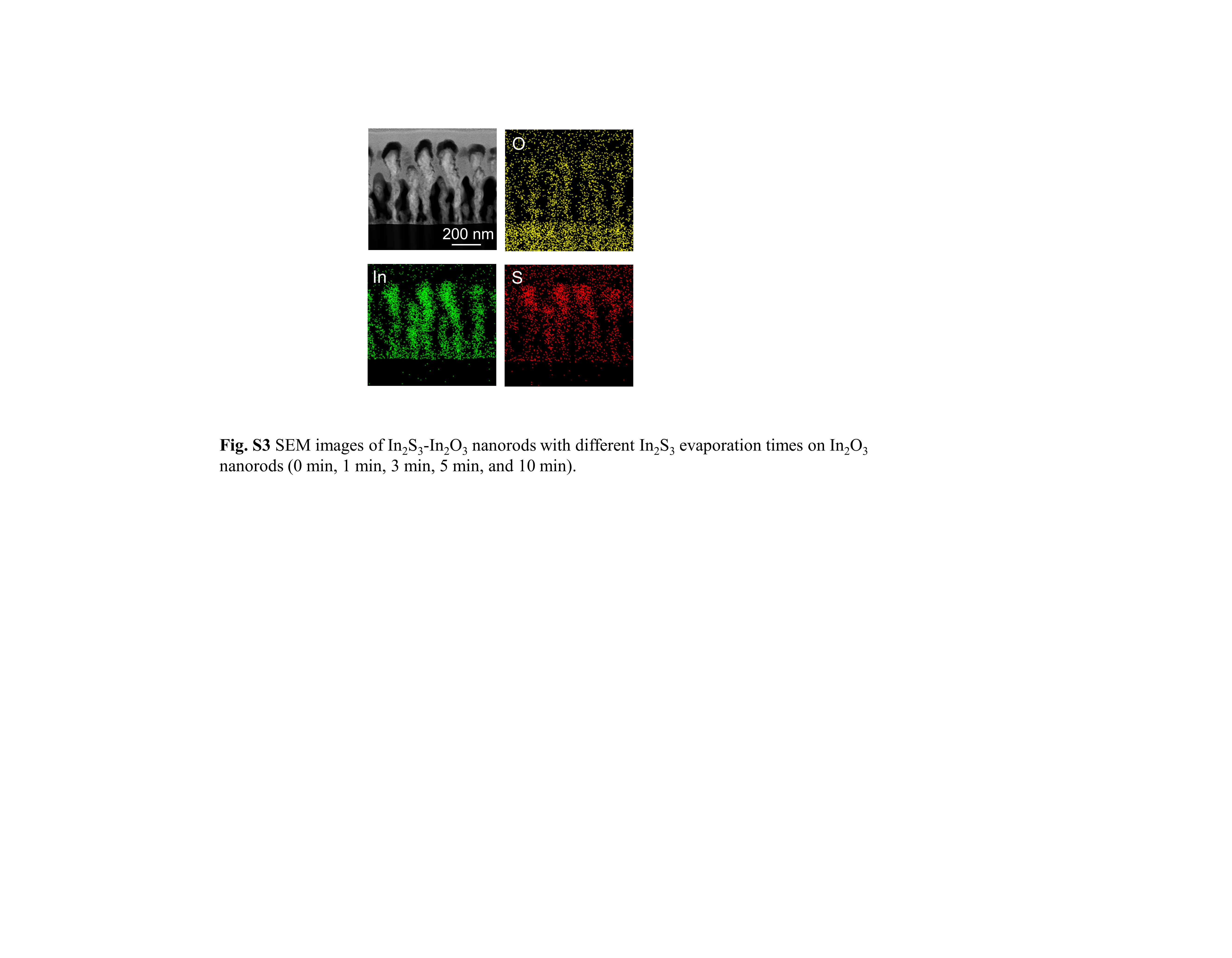
**

**Figure S6**. EDS analysis of 3-ISO to O, In, and S elements.


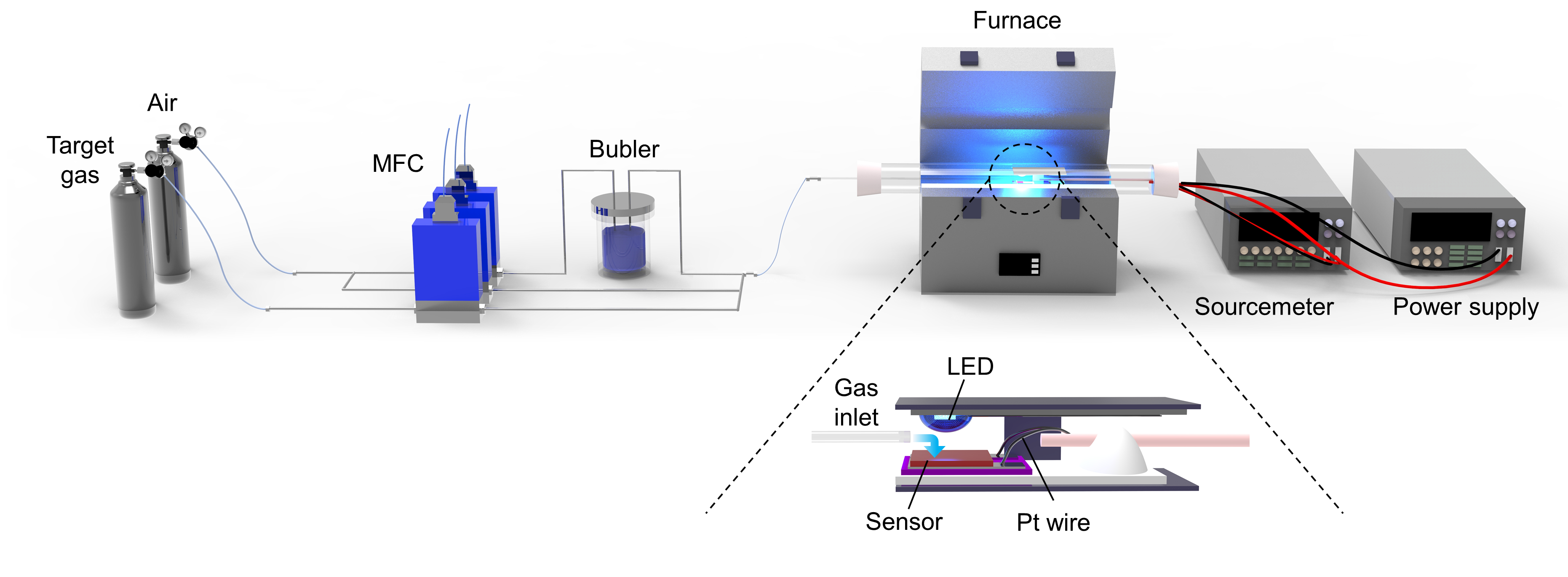


**Figure S7**. Schematic illustration of the gas measurement system.


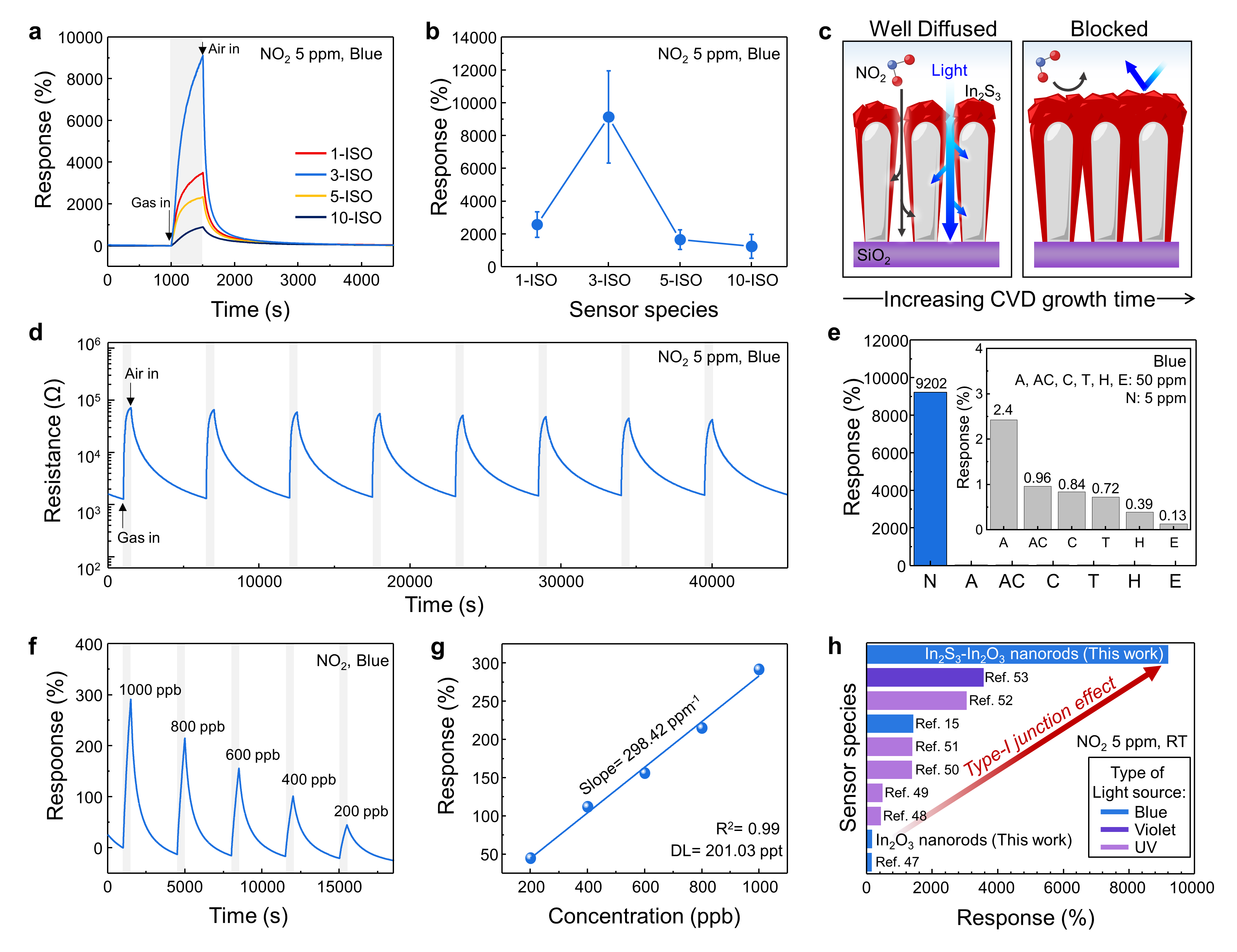


**Figure S8.** (a) Response curves and (b) response plots to 5 ppm of NO_2_ for ISO nanorods with different evaporation times under blue light illumination at room temperature. (c) Schematic illustration of the interpretation of the volcano-shaped response of ISO with different evaporation times.

To optimize the deposition time of In_2_S_3_ on In_2_O_3_, the gas sensing properties of ISO samples were evaluated at various In_2_S_3_ deposition times for 5 ppm of NO_2_ under blue light illumination at room temperature (Figure S8a). The gas response increased with the increasing deposition time of In_2_S_3_ nanocrystals by up to 3 minutes, but decreased with the excessive growth beyond 5 minutes (Figure S8b). During the early deposition stages, the In_2_S_3_ layer on In_2_O_3_ enhanced light activation, thereby improving the reactivity to NO_2_ gas. However, with excessive deposition, the porosity of the ISO heterostructure decreased, limiting the diffusion of gas and light to the lower layers of the ISO (Figure S8c and S2). Thus, the 3-ISO sample exhibited the highest response, attributed to its optimal structure with sufficient In_2_S_3_ coverage, allowing effective gas and light diffusion.

|  | **Light sources**  **Sensor species** | **Dark** | **Red** | **Green** | **Blue** | **UV** |
| --- | --- | --- | --- | --- | --- | --- |
| **Response time** | **In_2_S_3_-In_2_O_3_** | 430 s | 404 s | 386 s | 372 s | 318 s |
|  | **In_2_O_3_** | 450 s | 459 s | 423 s | 409 s | 56 s |
| **Recovery time** | **In_2_S_3_-In_2_O_3_** | not fully recovered | 1925 s | 850 s | 214 s | 571 s |
|  | **In_2_O_3_** | not fully recovered | not fully recovered | 2227 s | 2207 s | 870 s |

**Table S1.** Response and recovery times of 3-ISO and In_2_O_3_ nanorods under various light sources.


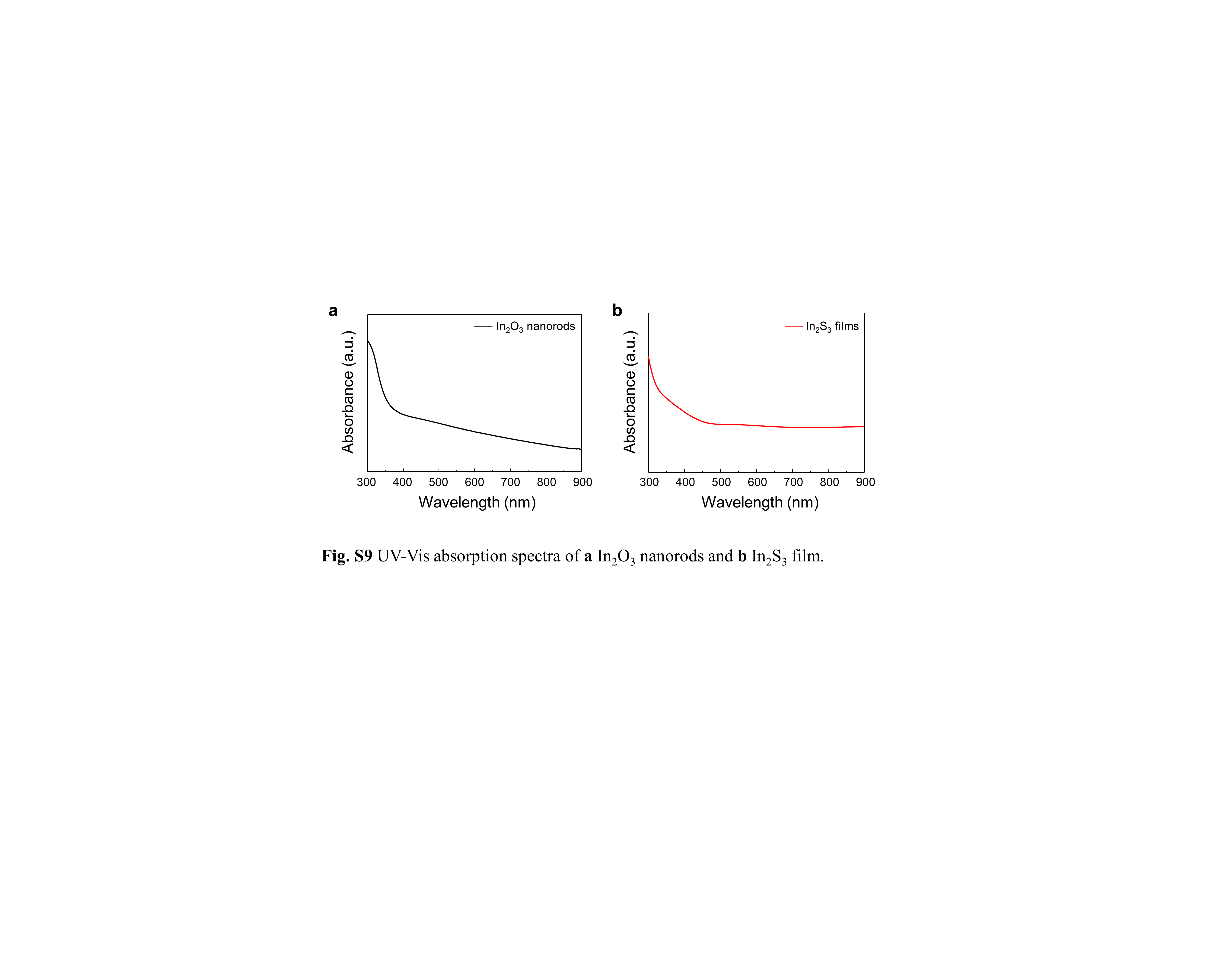


**Figure S9.** UV-vis absorption spectra of (a) In_2_O_3_ nanorods and (b) In_2_S_3_ film.


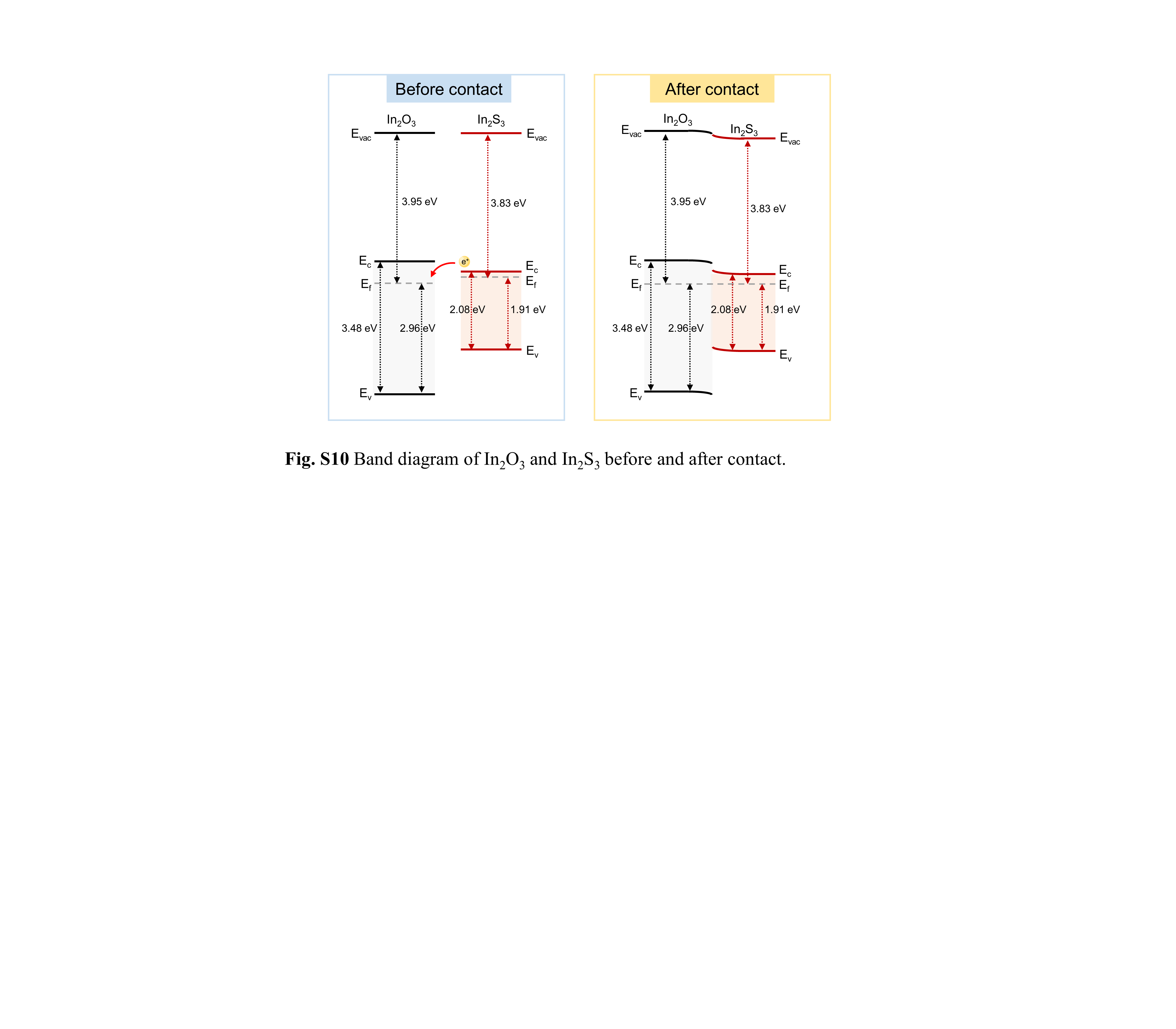


**Figure S10.** The energy band diagram of In_2_O_3_ and In_2_S_3_ before and after contact.


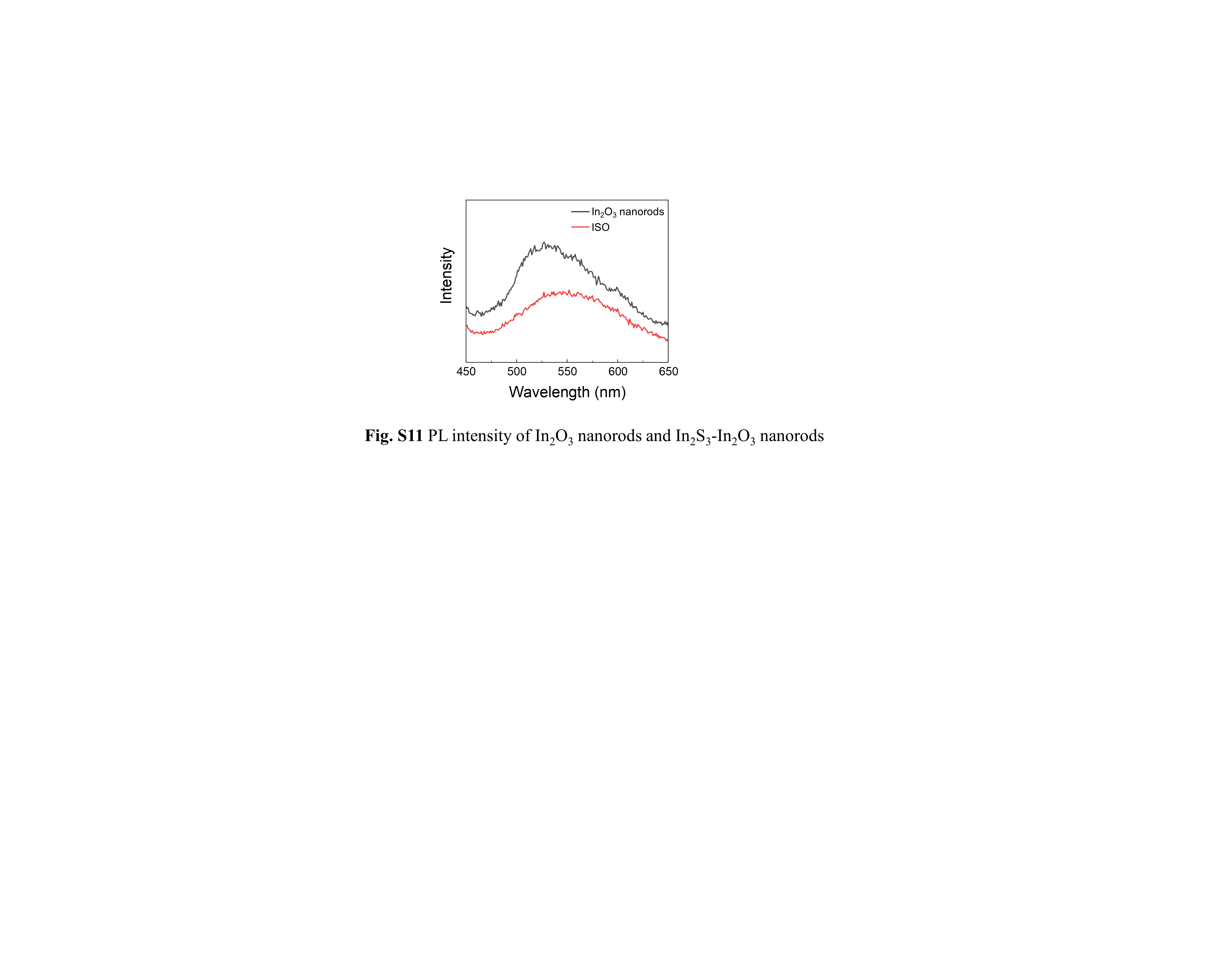


**Figure S11.** PL intensity of In_2_O_3_ NRs and ISO heterostructure.


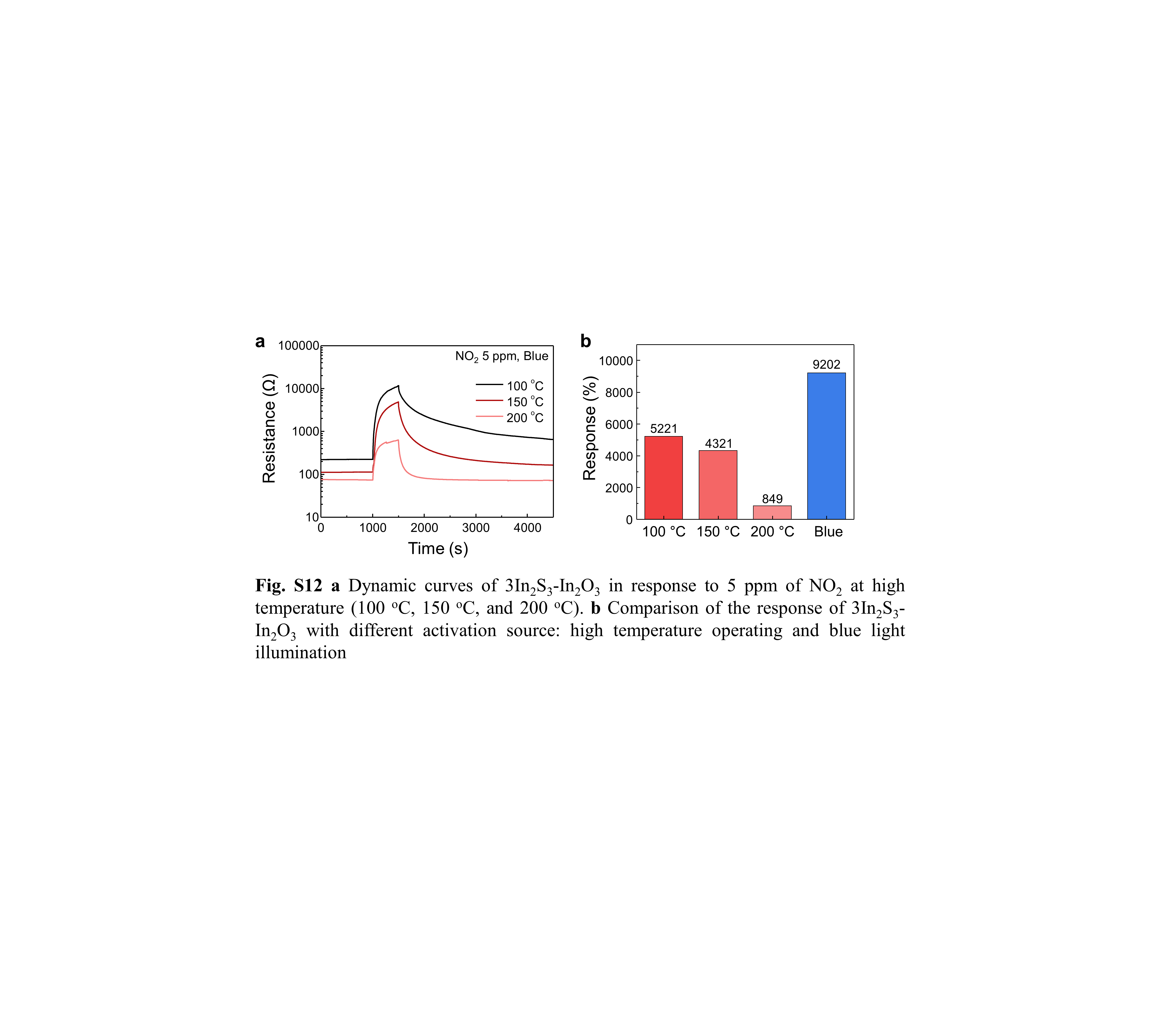


**Figure S12.** (a) Dynamic curves of 3-ISO in response to 5 ppm of NO_2_ at high temperatures (100, 150, and 200 ^°^C). (b) Comparison of the response of 3-ISO with different activation sources: high temperature operating and blue light illumination.


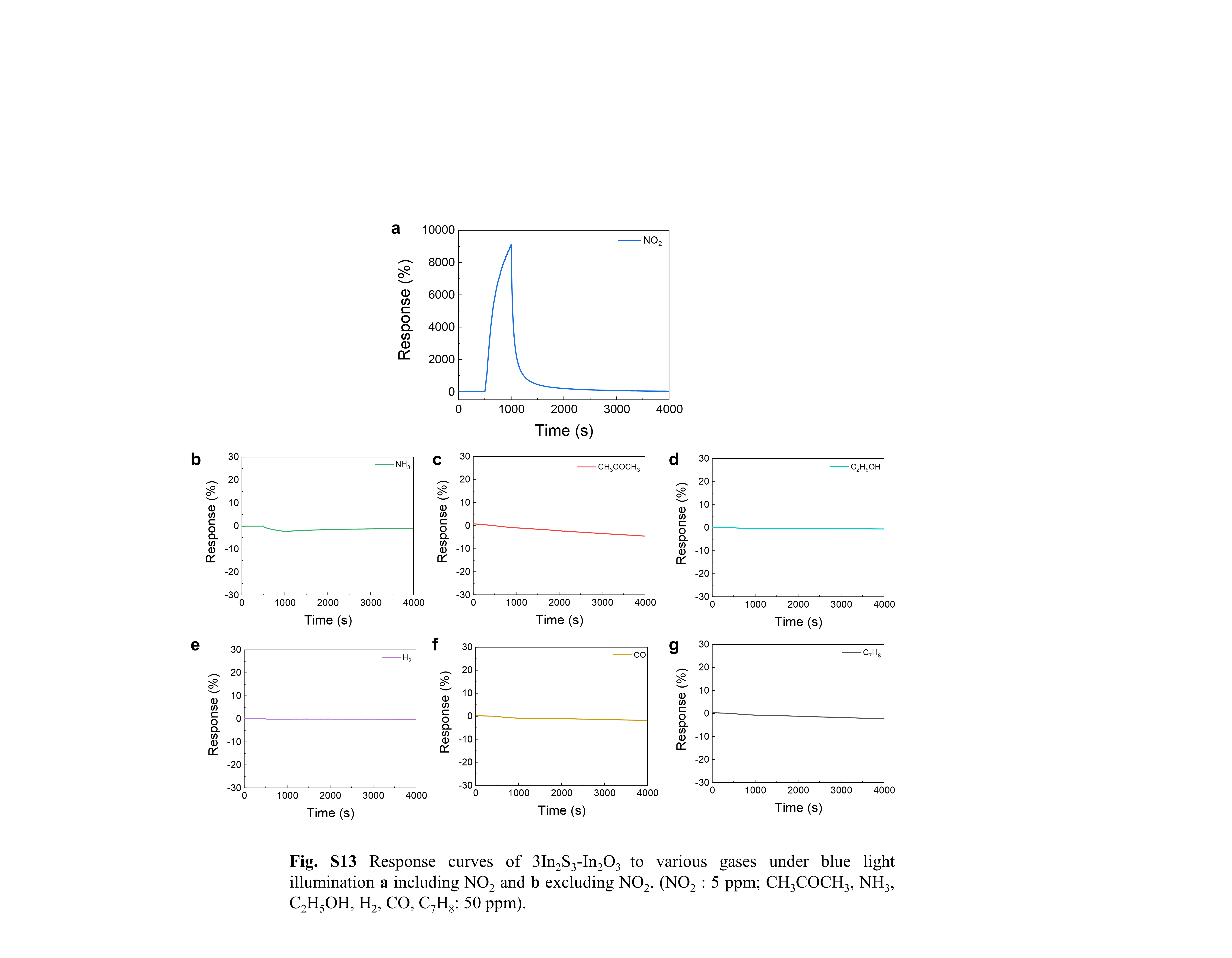


**Figure S13.** Response curves of 3-ISO to various gases under blue light illumination (a) including (a) NO_2_, (b) NH_3_, (c) CH_3_COCH_3_, (d) C_2_H_5_OH, (e) H_2_, (f) CO, and (g) C_7_H_8_. (NO_2_: 5 ppm; CH_3_COCH_3_, NH_3_, C_2_H_5_OH, H_2_, CO, C_7_H_8_: 50 ppm).


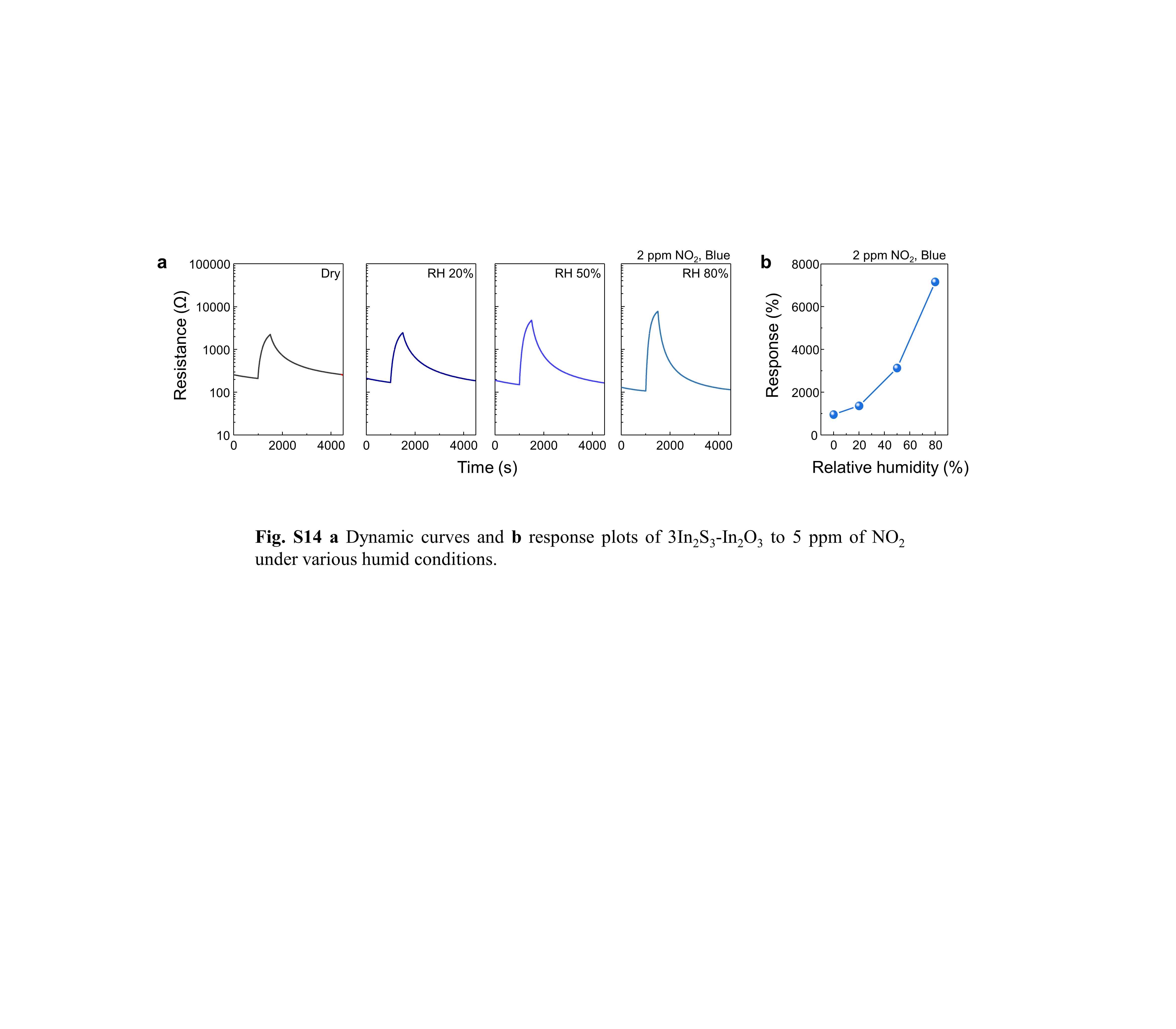


**Figure S14** (a) Dynamic curves and (b) response plots of 3-ISO to 2 ppm of NO_2_ under various humid conditions.


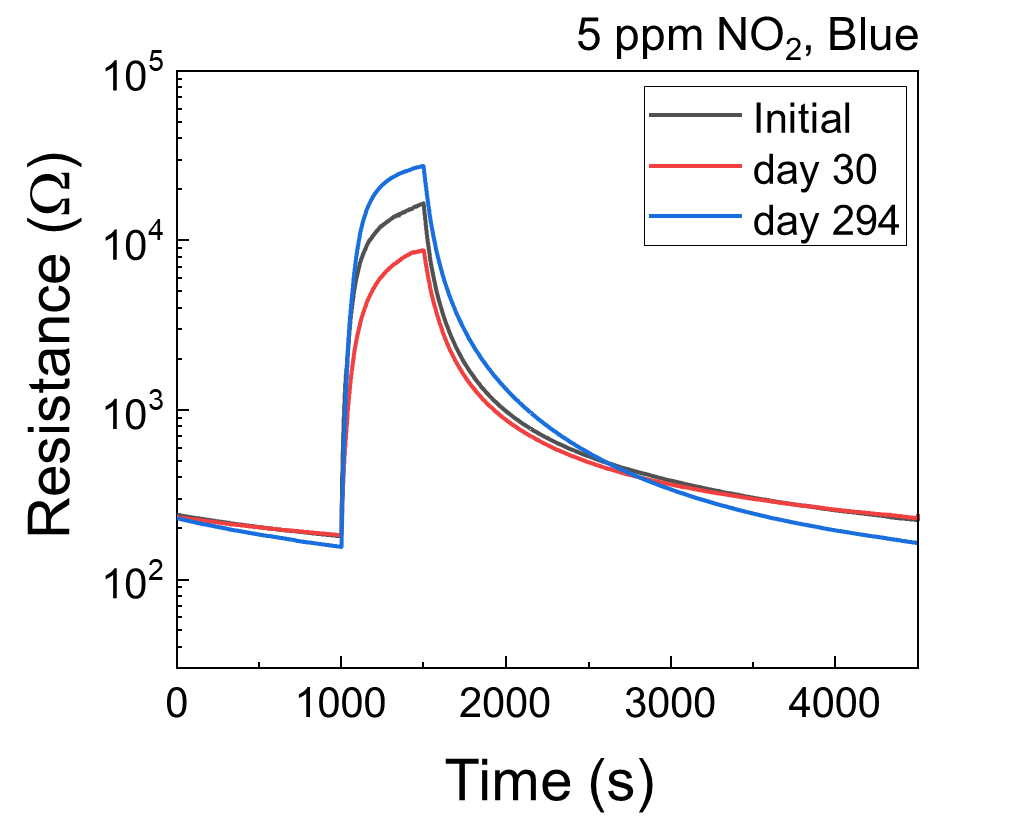


**Figure S15.** Comparison of the dynamic curves of 3-ISO at the initial state and after 30 and 294 days for the 5 ppm of NO_2_.

**Table S2.** Comparison of sensing properties of light-activated NO_2_ gas sensors.

| **Materials** | **Light source** | **Concentration (ppm)** | **Response (%)** | **Ref** |
| --- | --- | --- | --- | --- |
| **In_2_S_3_/In_2_O_3_ NRs** | **Blue**  **(450-455 nm)** | **5** | **9202** | **This work** |
| Ag decorated ZnO NPs. | Blue (470 nm) | 5 | 155 | [R6] |
| WS_2_/SnO_2_ | UV  (365 nm) | 5 | 430 | [R7] |
| SnO_2_ monolayer | UV  (365 nm) | 5 | 481 | [R8] |
| S-doped In_2_O_3_ | UV  (380 nm) | 10 | 1380 | [R9] |
| ZnO/In_2_O_3_nanofiber | UV  (365 nm) | 5 | 1400 | [R10] |
| SnS_2_ nanoflowers | Blue  (450-455 nm) | 5 | 1428 | [R11] |
| TiO_2_ thin-shell | UV | 5 | 3058 | [R12] |
| TiO_2_-In_2_O_3_ | Violet  (395-405 nm) | 5 | 3580 | [R13] |
| ZnO nanowires | UV (365 nm) | 1 | 708 | [R14] |
| Fe_2_O_3_/Cu_3_(HHTP)_2_ | Blue | 5 | 89.4 | [R15] |
| CVD grown MoS_2_ | UV | 5 | 11.5 | [R16] |
| Graphene | UV | 1 | 20 | [R17] |
| Au-decorated MoS_2_ | White light | 10 | 27.52 | [R18] |
| Close-packed SnO_2_ monolayer film | UV  (365 nm) | 5 | 1053 | [R19] |
| Au-ZnO nanosheets | UV  (365 nm) | 5 | 455 | [R20] |
| SnS_2_ nanosheets | Green  (520-550 nm) | 8 | 1510 | [R21] |
| CdS nanoflakes | Green | 5 | 182 | [R22] |
| ZnO nanowires | UV  (390 nm) | 1 | 388 | [R23] |
| ZnO/PbS | NIR  (831 nm) | 10 | 490 | [R24] |
| Bi_2_O_3_/ZnO | UV  (254 nm) | 5 | 665 | [R25] |


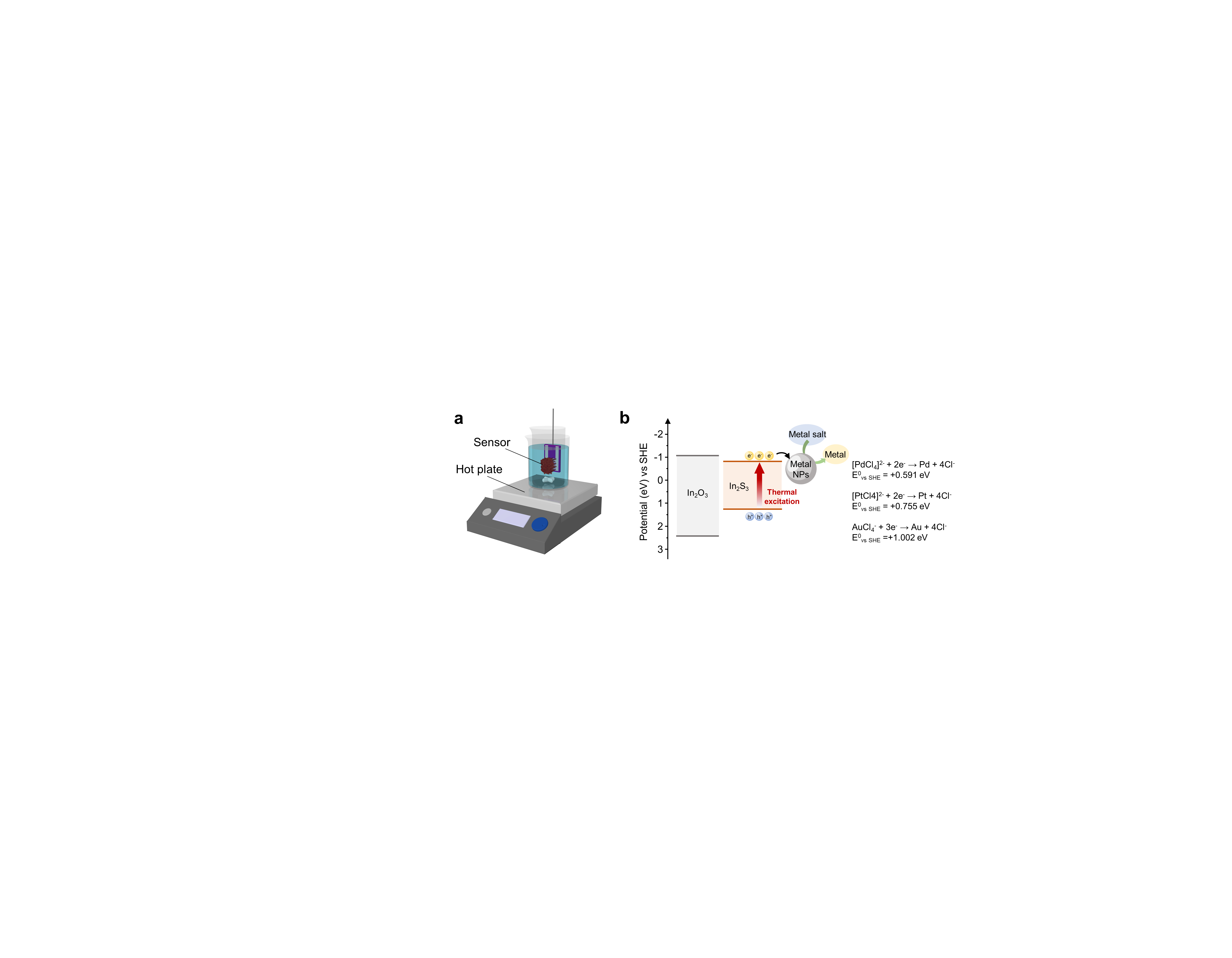


**Figure S16.** (a) Schematic illustration of the deposition of noble metal using the solution process. (b) Mechanism of noble metal decoration on In_2_S_3_ surface.


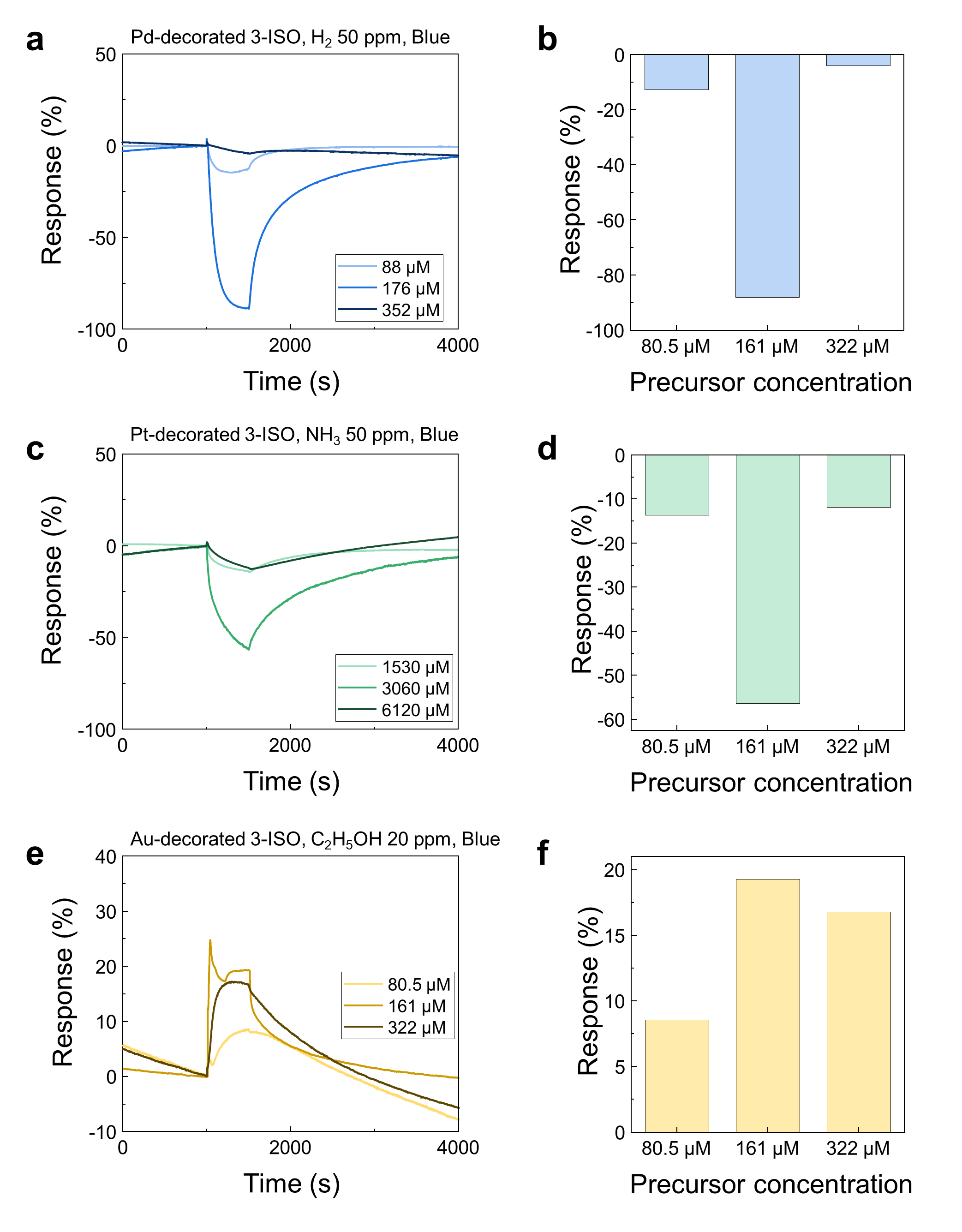


**Figure S17.** (a) Response curves and (b) response plot for 50 ppm of H_2_ at different Pd-precursor concentrations in the synthesis of Pd-decorated 3-ISO. (c) Response curves and (d) response plot for 50 ppm of NH_3_ at different Pt-precursor concentrations in the synthesis of Pd-decorated 3-ISO. (e) Response curves and (f) response plot for 20 ppm of C_2_H_5_OH at different Au-precursor concentrations in the synthesis of Au-decorated 3-ISO.


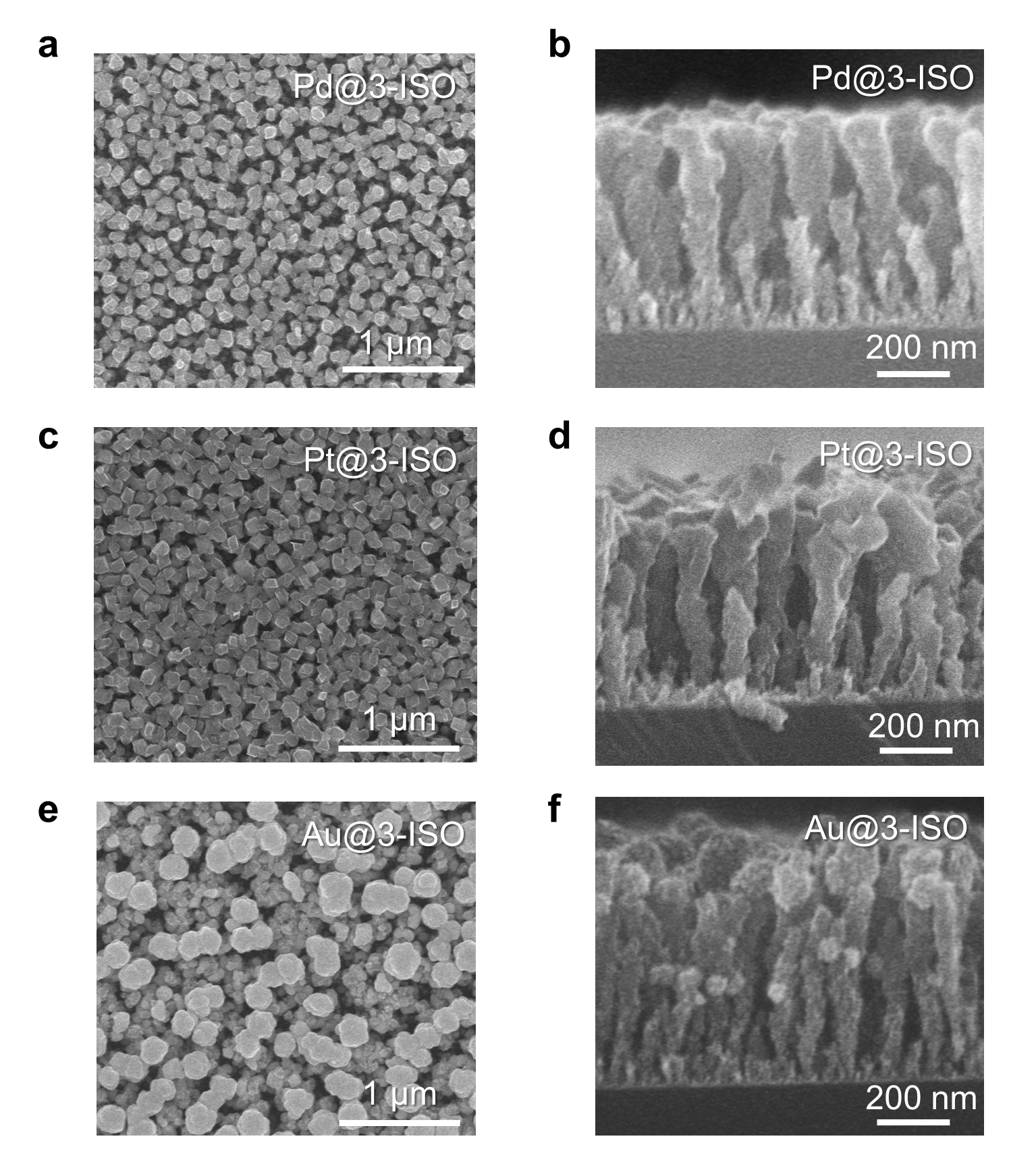


**Figure S18.** (a) SEM and (b) cross-sectional SEM images of Pd@3-ISO. (c) SEM and (d) cross-sectional SEM of Pt@3-ISO. (e) SEM and (f) cross-sectional SEM images of Au@3-ISO.

**
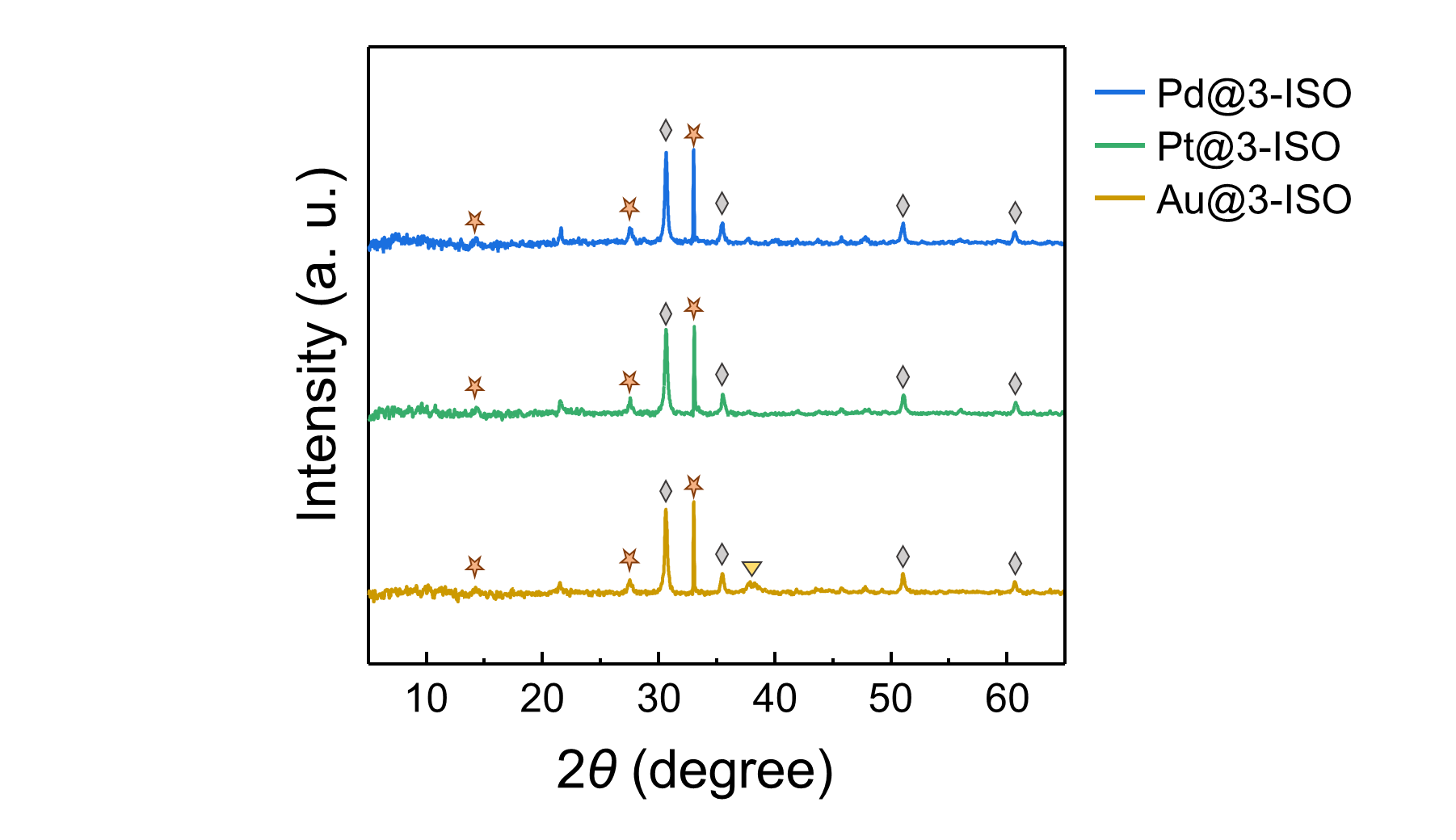
**

**Figure S19.** XRD spectrum of Pd@3-ISO, Pt@3-ISO, and Au@3-ISO. Diamonds indicates In_2_O_3_ peaks, stars indicate In_2_S_3_ peaks, and triangle indicate Au peak.


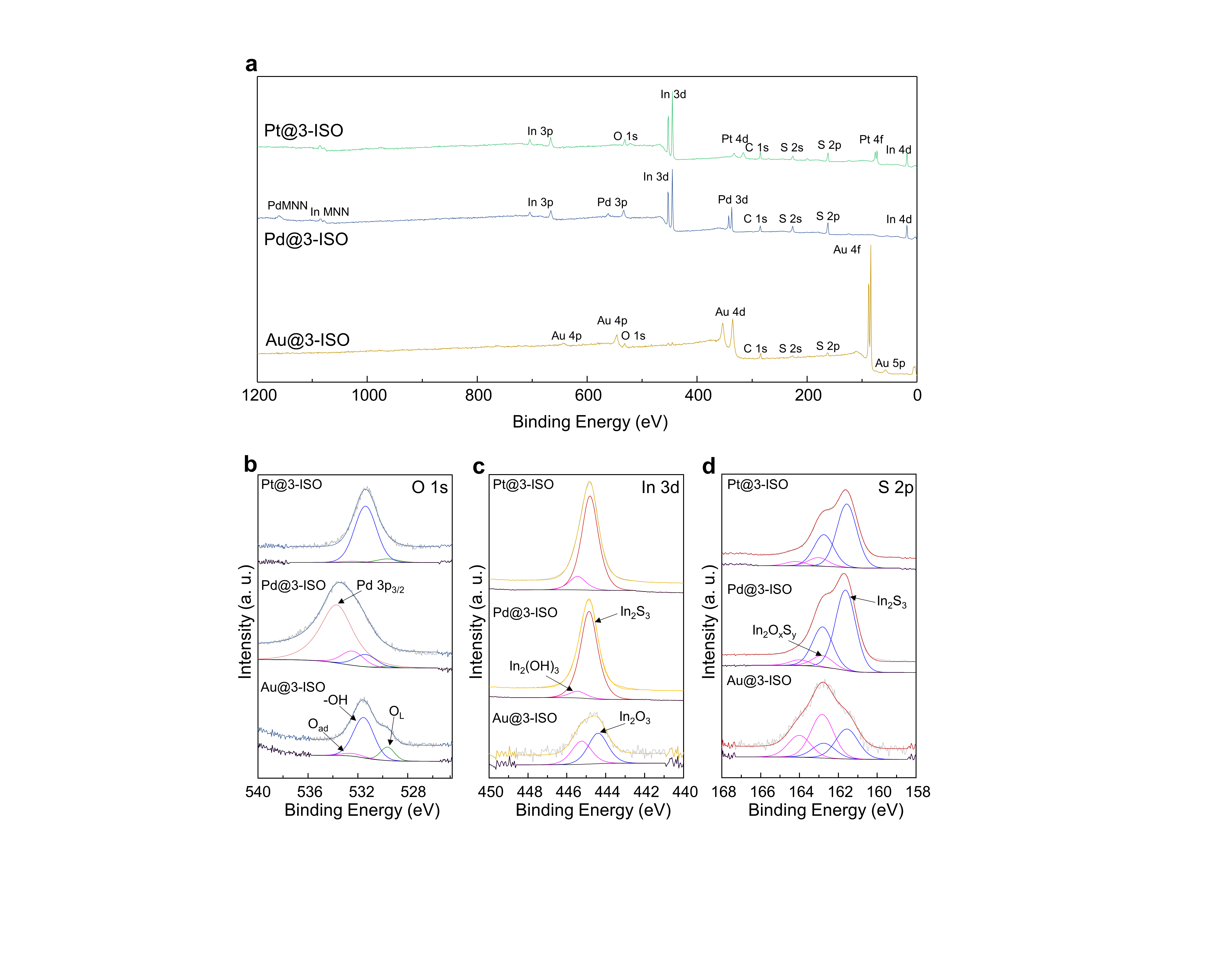


**Figure S20**. (a) XPS survey spectra of Pt@3-ISO, Pd@3-ISO, and Au@3-ISO. (b) O 1s, (c) In 3d, and (d) S 2p XPS spectra of Pt@3-ISO, Pd@3-ISO, and Au@3-ISO.

XPS analysis confirmed the successful and uniform decoration of noble metals (Pt, Pd, Au) onto ISO heterostructures. Survey spectra clearly revealed characteristic peaks for In and O across all samples, confirming the presence of the ISO heterostructure. Notably, the appearance of distinct S peaks exclusively in metal-decorated samples indicated effective deposition of the additional In_2_S_3_ layer during decoration. Deconvolution of the O 1s spectrum exhibited a marked decrease in lattice oxygen (530.3 eV) upon noble metal decoration, accompanied by enhanced signals corresponding to oxygen vacancies (531.8–531.9 eV) and surface-adsorbed oxygen species (532.6 eV), reflecting lattice rearrangements and surface modifications induced by noble metal interactions.^[R26]^

In the In 3d_5/2_ spectra, peaks corresponding to In_2_O_3_ (444.6 eV) and In_2_S_3_ (445.0 eV) were simultaneously observed, confirming the coexistence of oxide and sulfide phases.^[R27]^ The prominence of In_2_S_3_ peaks was especially evident in Pt@3-ISO and Pd@3-ISO samples, underscoring the enhanced deposition of In_2_S_3_ promoted by these metals. Conversely, Au@3-ISO maintained a relatively balanced coexistence, indicating effective but comparatively moderate sulfur incorporation. In_2_S_3_ and In(OH)_3_ (445.3 eV) became dominant with prolonged deposition, demonstrating progressive surface enrichment by In_2_S_3_ layers.^[R28]^

The S 2p spectra further validated the successful deposition and formation of In_2_S_3_, exhibiting clear peaks at 161.7 eV (In_2_S_3_) and minor peaks at 163.2–163.4 eV corresponding to intermediate mixed oxysulfide (InO_x_S_y_) phases.^[R29]^ The enhanced In_2_S_3_ peak intensity, particularly notable in Pt@3-ISO and Pd@3-ISO, strongly supports significant and uniform sulfur incorporation. Meanwhile, Au@3-ISO showed effective sulfide incorporation, preserving a more oxide-rich surface.^[R30]^


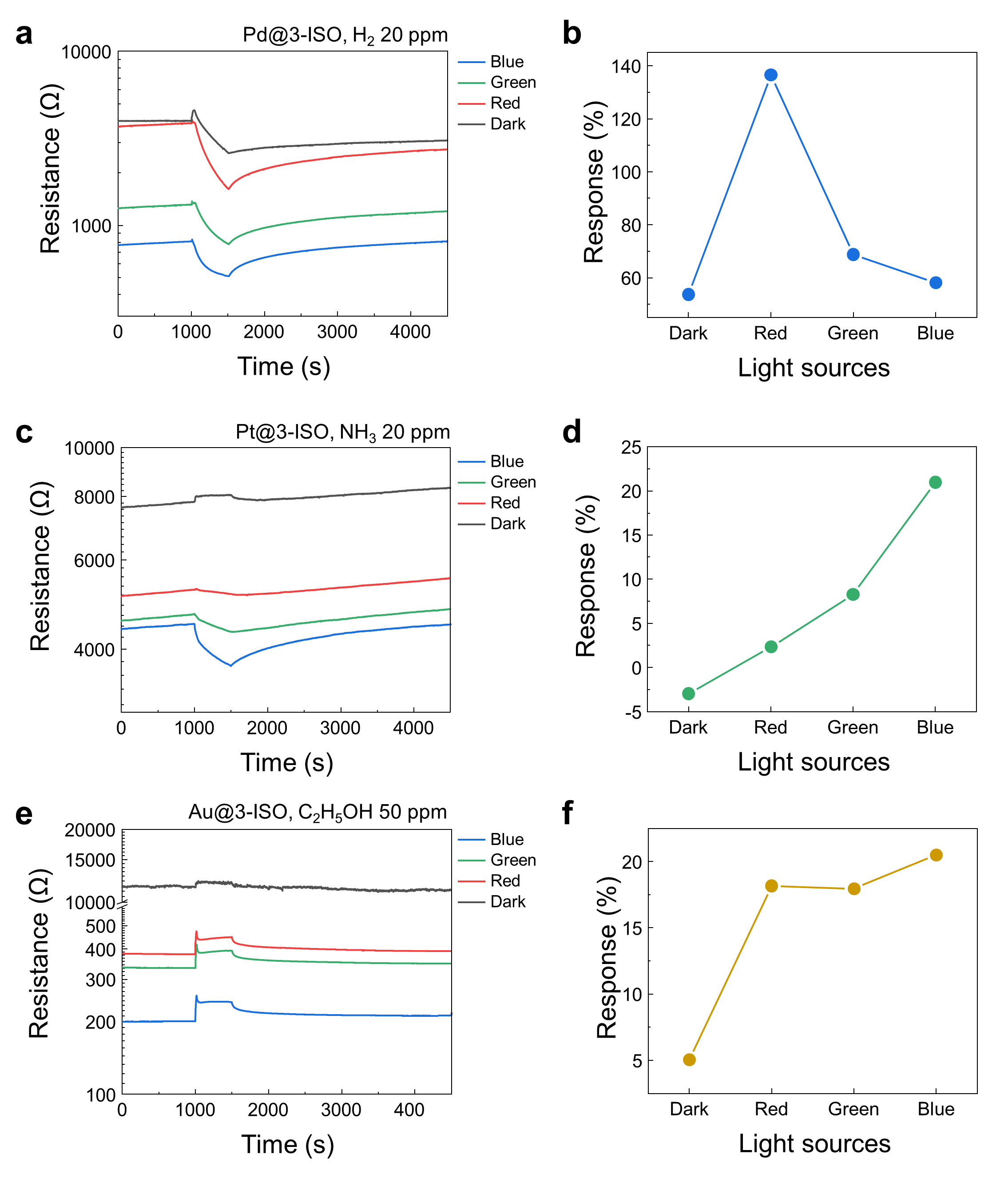


**Figure S21.** (a) Dynamic curves and (b) response plot of Pd@3-ISO to 20 ppm of H_2_ under dark conditions and red, green, and blue light illumination. (c) Dynamic curves and (d) response plot of Pt@3-ISO to 20 ppm of NH_3_ under dark conditions and red, green, and blue light illumination. (e) Dynamic curves and (f) response plot of Au@3-ISO to 50 ppm of C_2_H_5_OH under dark conditions and red, green, and blue light illumination.

The responses of Pd@3-ISO, Pt@3-ISO, and Au@3-ISO were evaluated under dark, red, green, and blue light illumination. For Pd@3-ISO, blue light illumination exhibited the shortest response time and complete recovery to the baseline, although the response magnitude was not the highest. Under dark, red, and green illumination, Pd@3-ISO showed large response amplitudes but sluggish kinetics and incomplete recovery. For Pt@3-ISO and Au@3-ISO, blue light illumination exhibits the highest response along with fast and reversible behavior. Accordingly, blue-illuminated operation was selected for the 2 × 2 gas sensor array comprising 3-ISO, Pd@3-ISO, Pt@3-ISO, and Au@3-ISO, as it provides the most favorable sensing characteristics.


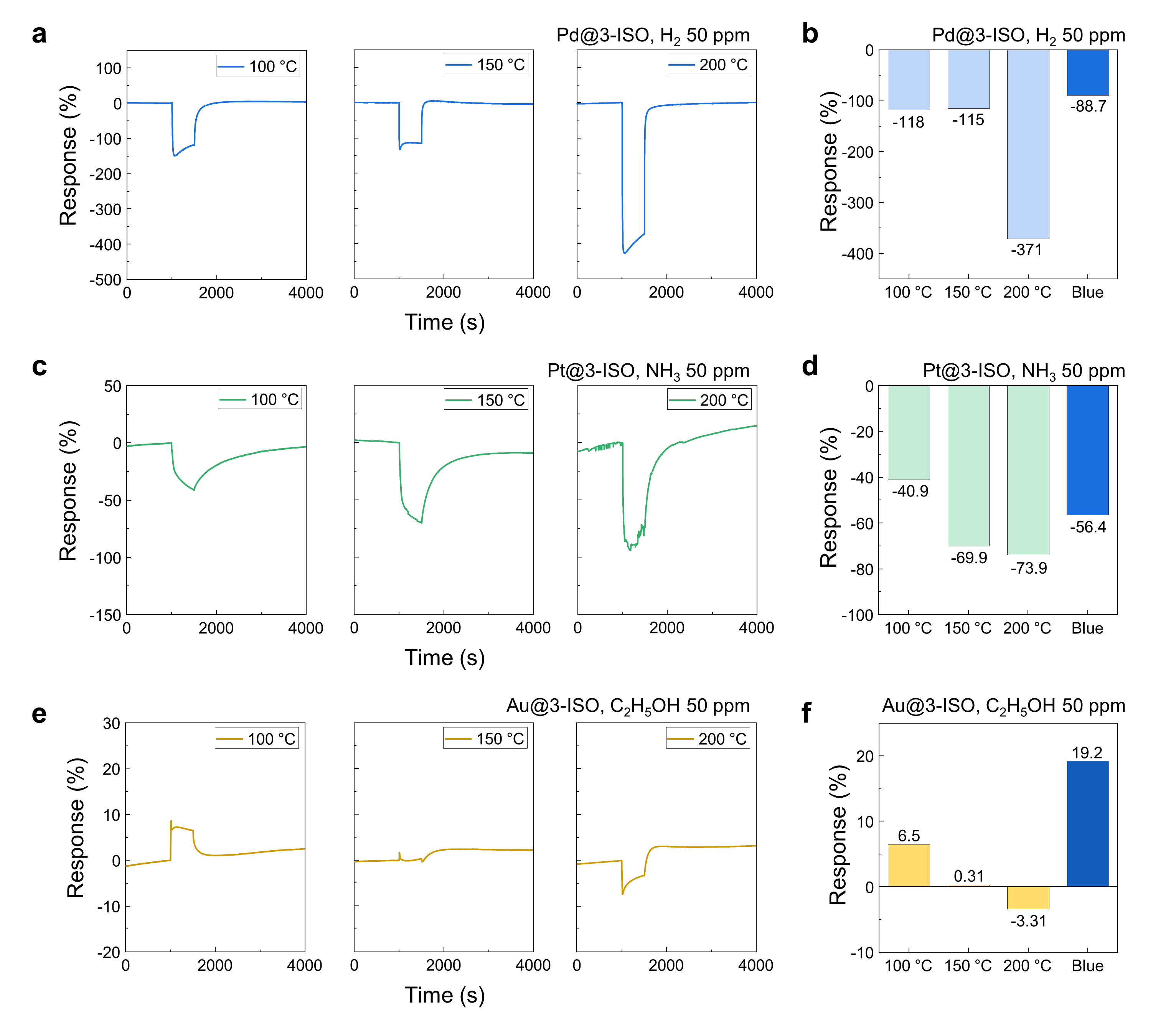


**Figure S22.** (a) Response curves of Pd@3-ISO to 50 ppm of H_2_ at high temperature (100 ^°^C, 150 ^°^C, and 200 ^°^C) and (b) comparison with blue light illumination. (c) Response curves of Pt@3-ISO to 50 ppm of NH_3_ at high temperatures (100 ^°^C, 150 ^°^C, and 200 ^°^C) and (d) comparison with blue light illumination. (e) Response curves of Au@3-ISO to 50 ppm of C_2_H_5_OH at high temperatures (100 ^°^C, 150 ^°^C, and 200 ^°^C) and (f) comparison with blue light illumination.


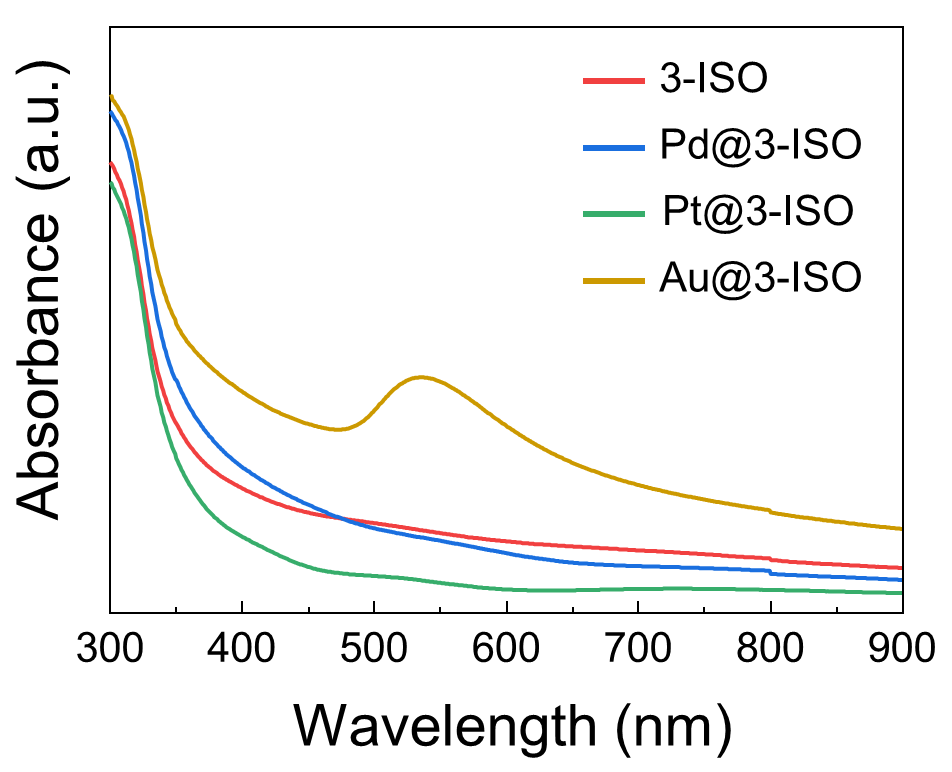


**Figure S23.** UV-vis absorption spectra of 3-ISO, Pd@3-ISO, Pt@3-ISO, and Au@3-ISO.

.


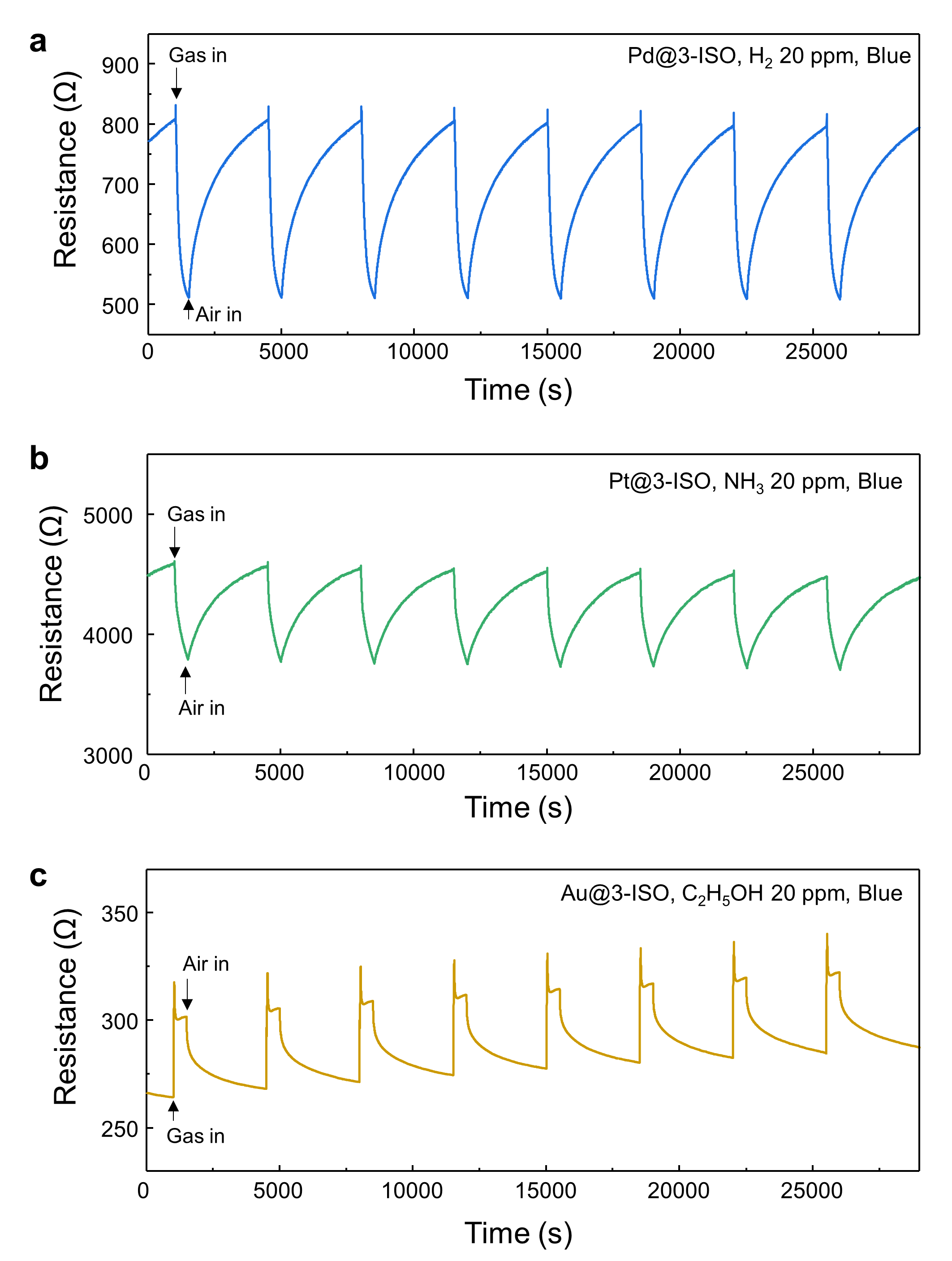


**Figure S24**. Reliability of (a) Pd@3-ISO to H_2_, (b) Pt@3-ISO to NH_3_, and (c) Au@3-ISO to C_2_H_5_OH to eight pulses under blue light illumination.


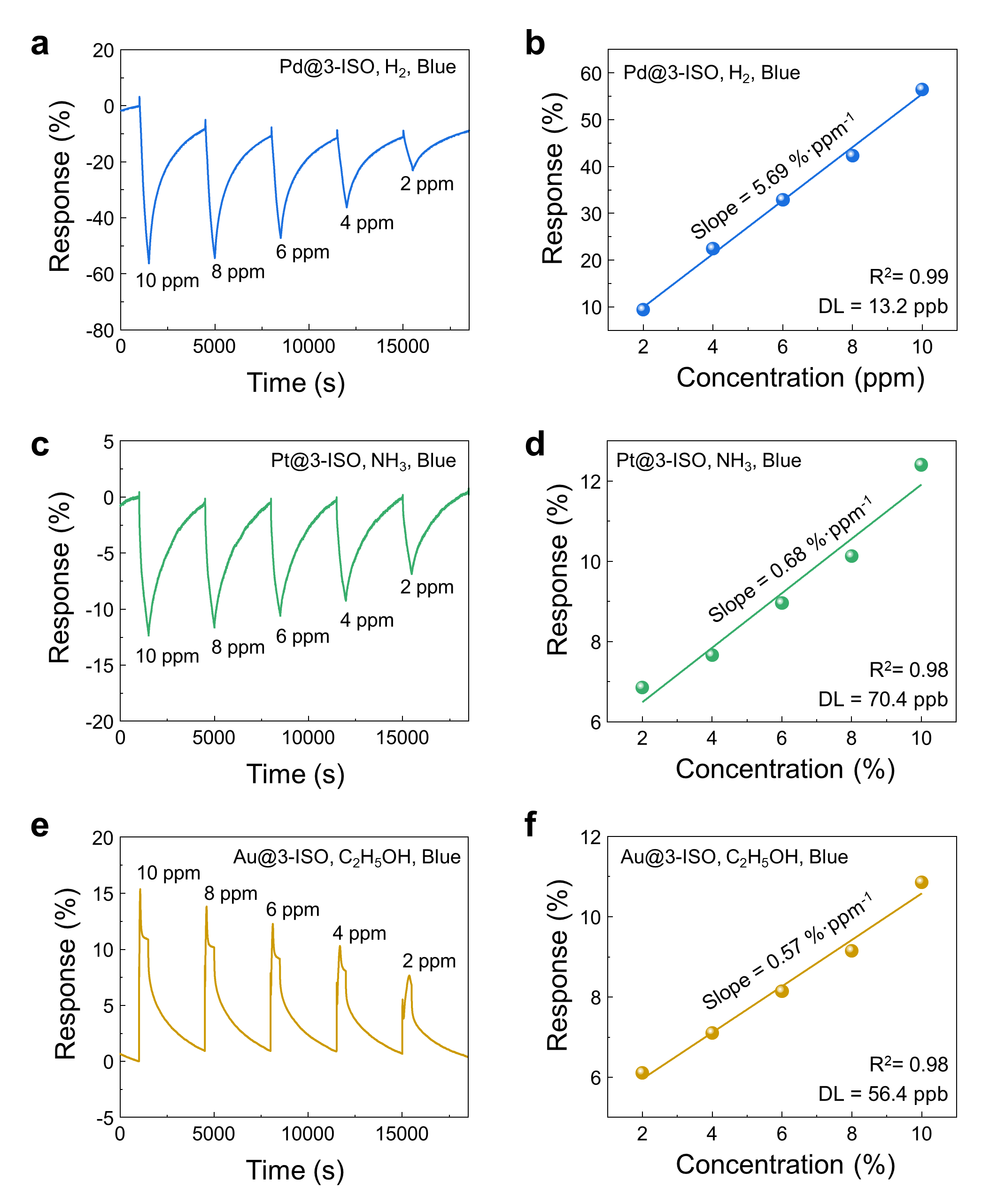


**Figure S25.** (a) Response curves and (b) response calibration of Pd@3-ISO to 2-10 ppm of H_2_ under blue light illumination. (c) Response curves and (d) response calibration of Pt@3-ISO to 2-10 ppm of NH_3_ under blue light illumination. (e) Response curves and (f) response calibration Au@3-ISO to 2-10 ppm of C_2_H_5_OH under blue light illumination.


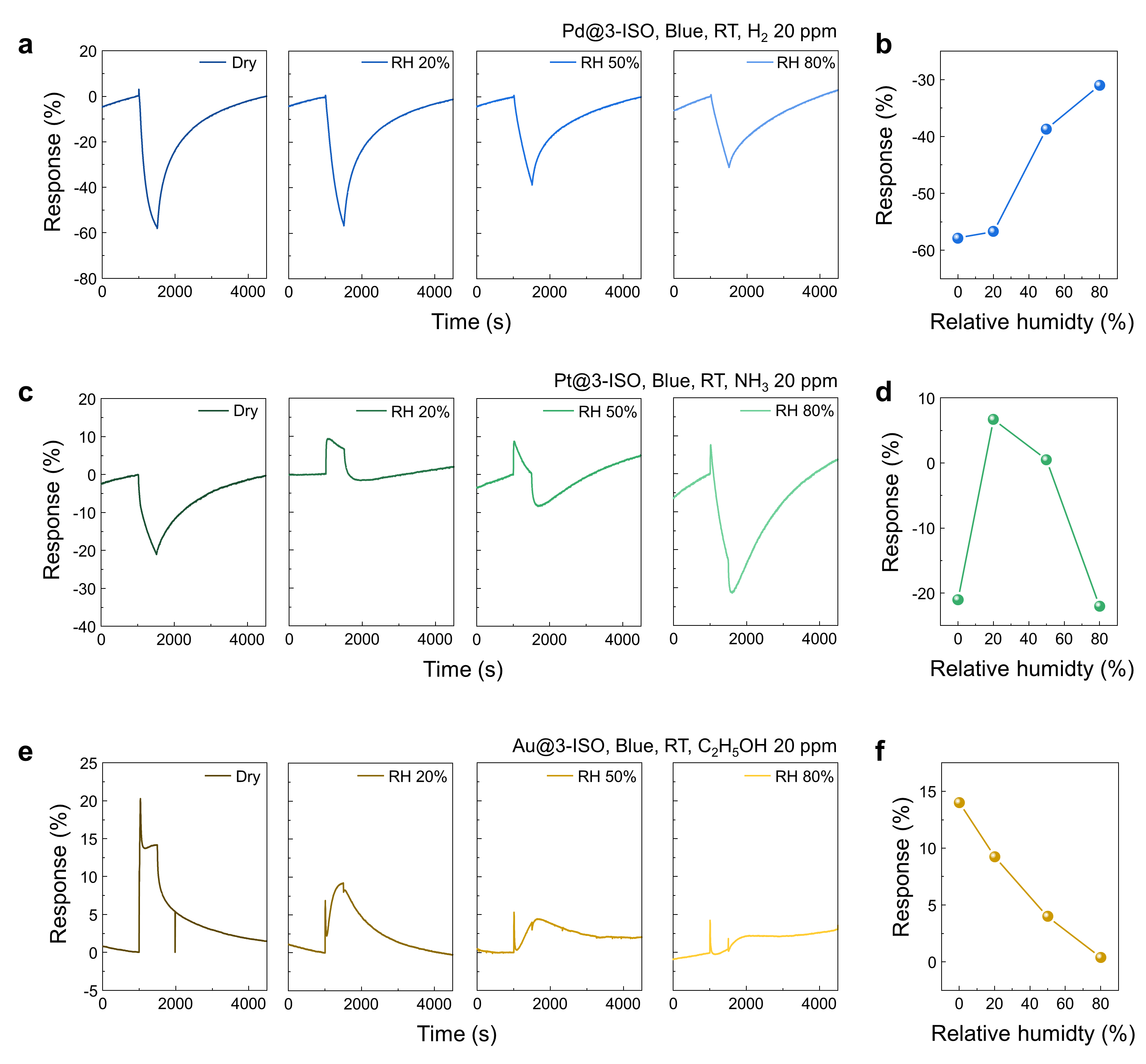


**Figure S26.** (a) Response curves and (b) response calibration of Pd@3-ISO to 20 ppm of H_2_ under dry air to RH 80%. (c) Response curves and (d) response calibration of Pt@3-ISO to 20 ppm of NH_3_ under dry air to RH 80%. (e) Response curves and (f) response calibration Au@3-ISO to 20 ppm of C_2_H_5_OH under dry air to RH 80%.

The response of Pd@3-ISO to 20 ppm of H_2_, Pt@3-ISO to NH_3_, and Au@3-ISO to C_2_H_5_OH was measured at RH values of 0, 20, 50, and 80% to assess sensing performance under humid conditions. For Pd@3-ISO, the response to H_2_ decreased with increasing RH (Figure S26a and b). Competitive adsorption of H_2_O on active sites reduces the surface coverage of oxygen species, which suppresses the reaction between H_2_ and adsorbed oxygen. For Pt@3-ISO, the response decreased at low RH and increased again at high RH (Figure S26c and d). At low RH, H_2_O adsorbs and forms hydroxyls that compete with oxygen adsorption, reducing the extent of NH_3_ oxidation.^[R31]^ At high RH, proton hopping via the Grotthuss mechanism occurs on a physisorbed H_2_O layer.^[R32-34]^ On the hydrated surface, NH_3_ reacts according to

NH_3(g)_ + H_2_O_(ad)_ → NH_4_^+^_(ad)_ + OH^-^_(ad)_ (R1)

which facilitates charge transfer and yields an enhanced response.^[R35-36]^ For Au@3-ISO, the response to C_2_H_5_OH decreases as RH increases (Figure S26e and f). Competitive adsorption of H_2_O on Au NPs hinders C_2_H_5_OH absorption and weakens the electronic-scattering driven resistance change, leading to a lower response.^[R37]^


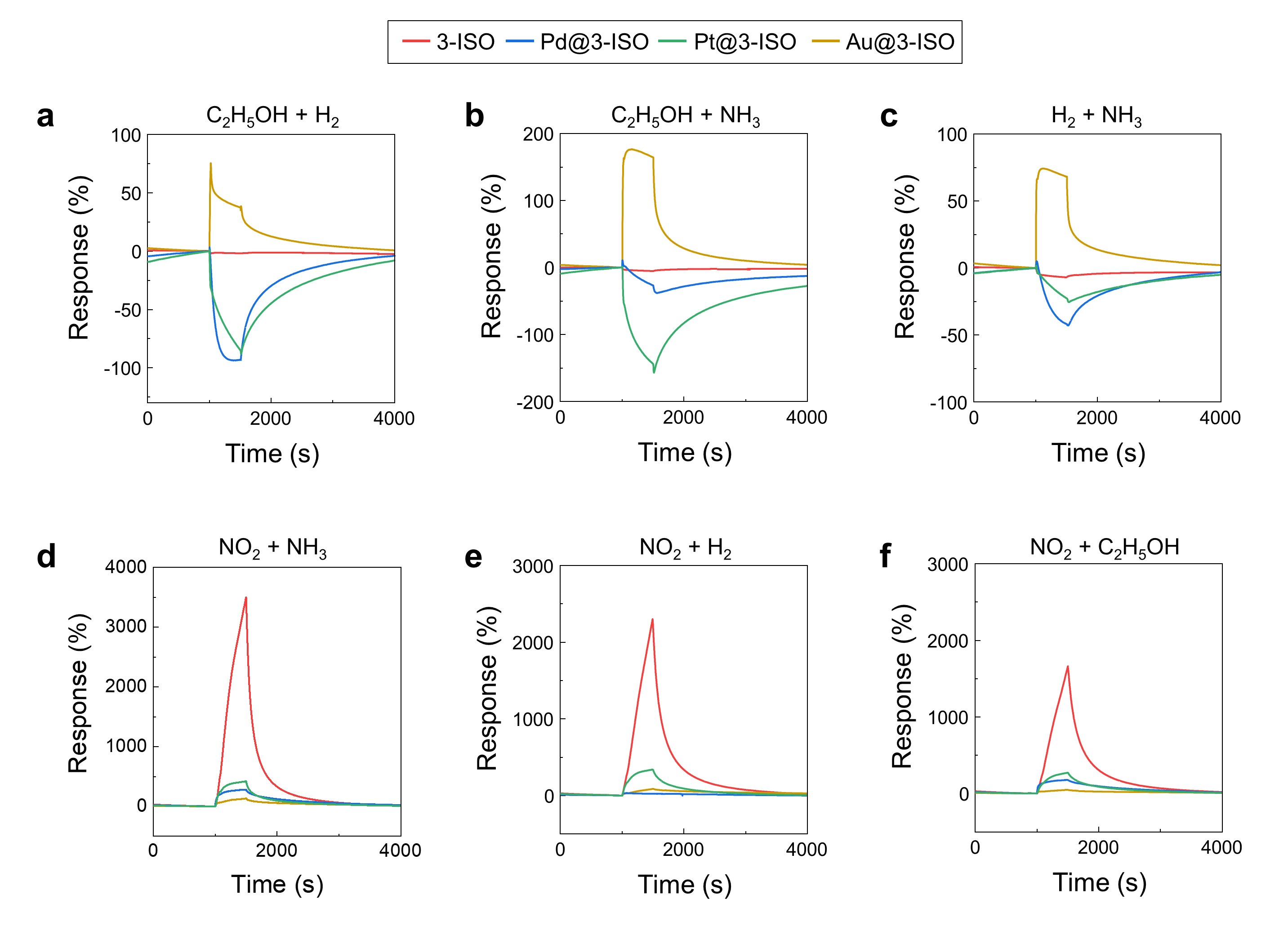


**Figure S27.** Response of 3-ISO, Pd@3-ISO, Pt@3-ISO, and Au@3-ISO to gas mixtures of (a) C_2_H_5_OH and H_2_, (b) C_2_H_5_OH and NH_3_, (c) H_2_ and NH_3_, (d) NO_2_ and NH_3_, (e) NO_2_ and H_2_, and (f) NO_2_ and C_2_H_5_OH under blue light illumination. Each gas concentration is 1 ppm for NO_2_ and 50 ppm for C_2_H_5_OH, H_2_, and NH_3_.

**
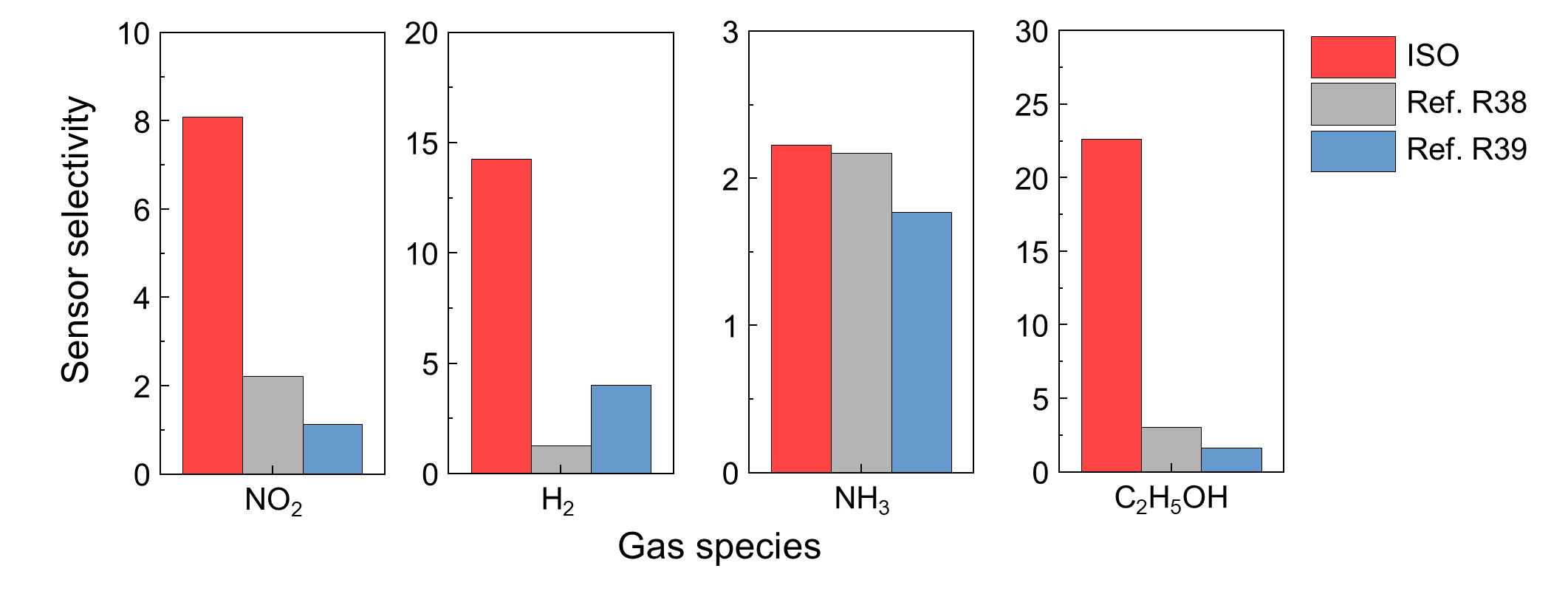
Figure. S28.** Comparison of sensor selectivity for ISO and other noble metal-decorated 2 × 2 sensor arrays^[R38-39]^ toward NO_2_, H_2_, NH_3_, and C_2_H_5_OH. Sensor selectivity is defined as the ratio of the highest sensor response to the second-highest response within each array.

**Reference**

[R1] D. A. Zatsepin, D. W. Boukhvalov, A. F. Zatsepin, L. Vines, D. Gogova, V. Y. Shur, A. A. Esin, *J. Mater. Sci.: Mater. Electron.* **2019**, 30, 18753.

[R2] Y. Wang, L. Zhu, Y. Liu, E. I. Vovk, J. Lang, Z. Zhou, P. Gao, S. Li, Y. Yang, *Appl. Surf. Sci.* **2023**, 631.

[R3] Z. Wan, M. Hu, B. Hu, T. Yan, K. Wang, X. Wang, *Catal. Sci. Technol.* **2020**, 10, 2893.

[R4] A. Askarinejad, M. Iranpour, N. Bahramifar, A. Morsali, *J. Exp. Nanosci.* **2010**, 5, 294.

[R5] Y. Yao, Z. Zhao, H. Cui, W. Dong, Z. Li, G. Liao, *J. Mater. Sci.: Mater. Electron.* **2023**, 34.

[R6] Q. Zhang, G. Xie, M. Xu, Y. Su, H. Tai, H. Du, Y. Jiang, *Sens. Actuators B Chem.* **2018**, 259, 269.

[R7] Y. Xia, L. Xu, S. He, L. Zhou, M. Wang, J. Wang, S. Komarneni, *Sens. Actuators B Chem.* **2022**, 364.

[R8] B. Liu, Y. Luo, K. Li, H. Wang, L. Gao, G. Duan, *ACS Appl. Mater. Interfaces* **2019**, 6.

[R9] Y. Song, B. Yang, Z. Ma, Y. Song, J. Sun, *Sens. Actuators B Chem.* **2023**, 379.

[R10] C. Han, X. Li, Y. Liu, X. Li, C. Shao, J. Ri, J. Ma, Y. Liu, *J. Hazard. Mater.* **2021**, 403, 124093.

[R11] T. H. Eom, S. H. Cho, J. M. Suh, T. Kim, T. H. Lee, S. E. Jun, J. W. Yang, J. Lee, S.-H. Hong, H. W. Jang, *J. Mater. Chem. A* **2021**, 9, 11168.

[R12] D. Cho, J. M. Suh, S. H. Nam, S. Y. Park, M. Park, T. H. Lee, K. S. Choi, J. Lee, C. Ahn, H. W. Jang, Y. S. Shim, S. Jeon, *Advanced Science* **2021**, 8, 2001883.

[R13] S. Park, S. Jeon, H. Kim, J. Philips, D. Oh, J. Ahn, M. Kim, C. Park, S. Hong, J. Kim, W. Jung, I. D. Kim, *Adv. Funct. Mater.* **2023**, 33.

[R14] J. Wang, Y. Shen, X. Li, Y. Xia, C. Yang, *Sens. Actuators B Chem.* **2019**, 298, 126858.

[R15] Y.-M. Jo, K. Lim, J. W. Yoon, Y. K. Jo, Y. K. Moon, H. W. Jang, J.-H. Lee, *ACS Central Science* **2021**, 7, 1176.

[R16] Y. Kang, S. Pyo, E. Jo, J. Kim, *Nanotechnology* **2019**, 30, 355504.

[R17] C.-M. Yang, T.-C. Chen, Y.-C. Yang, M. Meyyappan, *RSC advances* **2019**, 9, 23343.

[R18] J. Hu, X. Liu, J. Zhang, X. Gu, Y. Zhang, *Sens. Actuators B Chem.* **2023**, 382, 133505.

[R19] B. Liu, Y. Luo, K. Li, H. Wang, L. Gao, G. Duan, *ACS Appl. Mater. Interfaces* **2019**, 6, 1900376.

[R20] Y. Mun, S. Park, S. An, C. Lee, H. W. Kim, *Ceram. Int.* **2013**, 39, 8615.

[R21] D. Gu, X. Wang, W. Liu, X. Li, S. Lin, J. Wang, M. N. Rumyantseva, A. M. Gaskov, S. A. Akbar, *Sens. Actuators B Chem.* **2020**, 305, 127455.

[R22] H.-Y. Li, J.-W. Yoon, C.-S. Lee, K. Lim, J.-W. Yoon, J.-H. Lee, *Sens. Actuators B Chem.* **2018**, 255, 2963.

[R23] I. Cho, Y. C. Sim, M. Cho, Y.-H. Cho, I. Park, *ACS sensors* **2020**, 5, 563.

[R24] R. Chen, J. Wang, Y. Xia, L. Xiang, *Sens. Actuators B Chem.* **2018**, 255, 2538.

[R25] S. Park, H. Ko, S. Lee, H. Kim, C. Lee, *Thin Solid Films* **2014**, 570, 298.

[R26] B.-R. Wang, L.-Y. Liu, G.-C. Guo, Y.-J. Bai, J.-C. Tu, R.-Z. Wang, *Appl. Surf. Sci.* **2022**, 584.

[R27] S. Li, K. Long, X. Sun, H. Yuan, W. Li, *Ceram. Int.* **2023**, 49, 24093.

[R28] H. Xu, Y. Wang, X. Dong, N. Zheng, H. Ma, X. Zhang, *Appl. Catal., B* **2019**, 257.

[R29] C. K. Nguyen, M. X. Low, A. Zavabeti, A. Jannat, B. J. Murdoch, E. Della Gaspera, R. Orrell-Trigg, S. Walia, A. Elbourne, V. K. Truong, C. F. McConville, N. Syed, T. Daeneke, *J. Mater. Chem. C* **2021**, 9, 11815.

[R30] F. Zhang, X. Li, Q. Zhao, A. Chen, *J. Phys. Chem. C* **2016**, 120, 19113.

[R31] B. P. Dhonge, S. S. Ray, B. Mwakikunga, *RSC Advances* **2017**, 7, 21703.

[R32] H.-J. Kim, J.-H. Lee, *Sens. Actuators B Chem.* **2014**, 192, 607.

[R33] P. Srinivasan, D. Prakalya, B. G. Jeyaprakash, *J. Alloys Compd.* **2020**, 819.

[R34] Q. Geng, X. Lin, R. Si, X. Chen, W. Dai, X. Fu, X. Wang, *Sens. Actuators B Chem.* **2012**, 174, 449.

[R35] S. L. Sibi, M. Rajkumar, M. Manoharan, J. Mobika, V. N. Priya, R. R. Kumar, *Analytica Chimica Acta* **2024**, 1287, 342075.

[R36] L. Liu, T. Fei, X. Guan, H. Zhao, T. Zhang, *Sens. Actuators B Chem.* **2021**, 334, 129625.

[R37] R. L. Freire, A. Kiejna, J. L. Da Silva, *Physical Chemistry Chemical Physics* **2016**, 18, 29526.

[R38] G. B. Nam, J.-E. Ryu, T. H. Eom, S. J. Kim, J. M. Suh, S. Lee, S. Choi, C. W. Moon, S. J. Park, S. M. Lee, *Nano-Micro Lett.* **2024**, 16, 261.

[R39] T. Kim, T. H. Lee, S. Y. Park, T. H. Eom, I. Cho, Y. Kim, C. Kim, S. A. Lee, M. J. Choi, J. M. Suh, I. S. Hwang, D. Lee, I. Park, H. W. Jang, *ACS Nano* **2023**, 17, 4404.
